# Supplementary material for: Abnormal expression of HOXD11 promotes the malignant behavior of glioma cells and leads to poor prognosis of glioma patients
Source: PeerJ. 2021 Feb 8;9:e10820. doi: 10.7717/peerj.10820 (PMC7877241; doi:10.7717/peerj.10820)
Supplement: Supplemental Information 1 [file peerj-09-10820-s001.docx]

**Table S1. Characteristics of patients with glioma based on the operating room.**

| **Characteristics** |  | **Number of cases** | **Percentages (%)** |
| --- | --- | --- | --- |
| Age | <=50 | 4 | 44.44 |
|  | >50 | 5 | 55.55 |
| Grade | WHO IV | 9 | 100 |
| Chemo status | Yes | 7 | 77.77 |
|  | No | 2 | 22.22 |
| PR type | Primary | 8 | 88.88 |
|  | Recurrent | 1 | 11.11 |
| Histology | GBM | 9 | 100 |
| Radio status | Yes | 8 | 88.88 |
|  | No | 1 | 11.11 |
| Gender | Male | 3 | 33.33 |
|  | Female | 6 | 66.66 |
