# Supplementary material for: Abnormal expression of HOXD11 promotes the malignant behavior of glioma cells and leads to poor prognosis of glioma patients
Source: PeerJ. 2021 Feb 8;9:e10820. doi: 10.7717/peerj.10820 (PMC7877241; doi:10.7717/peerj.10820)
Supplement: Supplemental Information 2 [file peerj-09-10820-s002.docx]

Table S2. Characteristics of patients with glioma based on CGGA

| **Characteristics** |  | **Number of cases** | **Percentages (%)** |
| --- | --- | --- | --- |
| Gender | Male | 307 | 40.99 |
|  | Female | 442 | 59.01 |
| Age | <=41 | 343 | 45.79 |
|  | >41 | 406 | 54.21 |
| Grade | WHO II | 218 | 29.11 |
|  | WHO III | 240 | 32.04 |
|  | WHO IV | 291 | 38.85 |
| PRS type | Primary | 502 | 67.02 |
|  | Recurrent | 222 | 29.64 |
|  | Secondary | 25 | 3.34 |
| Radio status | Yes | 625 | 83.44 |
|  | No | 124 | 16.56 |
| Chemo status | Yes | 520 | 69.43 |
|  | No | 229 | 30.57 |
| Histology | astrocytoma | 55 | 7.34 |
|  | Anaplastic astrocytoma | 39 | 5.21 |
|  | Anaplastic Oligodendroglioma | 22 | 2.94 |
|  | Anaplastic oligoastrocytoma | 80 | 10.68 |
|  | Glioblastoma | 176 | 23.50 |
|  | Oligodendroglioma | 35 | 4.67 |
|  | oligoastrocytoma | 95 | 12.68 |
|  | relapse astrocytoma | 20 | 2.67 |
|  | relapse Anaplastic astrocytoma | 36 | 4.81 |
|  | relapse Anaplastic Oligodendroglioma | 15 | 2.00 |
|  | relapse Anaplastic oligoastrocytoma | 48 | 6.41 |
|  | relapse Oligodendroglioma | 90 | 12.02 |
|  | relapse Oligodendroglioma | 4 | 0.53 |
|  | relapse oligoastrocytoma | 9 | 1.20 |
|  | Secondary relapse Oligodendroglioma | 25 | 3.34 |
| IDH mutation status | Mutant | 410 | 54.74 |
|  | Wildtype | 339 | 45.26 |
| 1p19q_codeletion_status | Non-codeletion | 155 | 20.69 |
|  | Codeletion | 594 | 79.31 |
