# Supplementary figures and images for: Abnormal expression of HOXD11 promotes the malignant behavior of glioma cells and leads to poor prognosis of glioma patients
Source: PeerJ. 2021 Feb 8;9:e10820. doi: 10.7717/peerj.10820 (PMC7877241; doi:10.7717/peerj.10820)

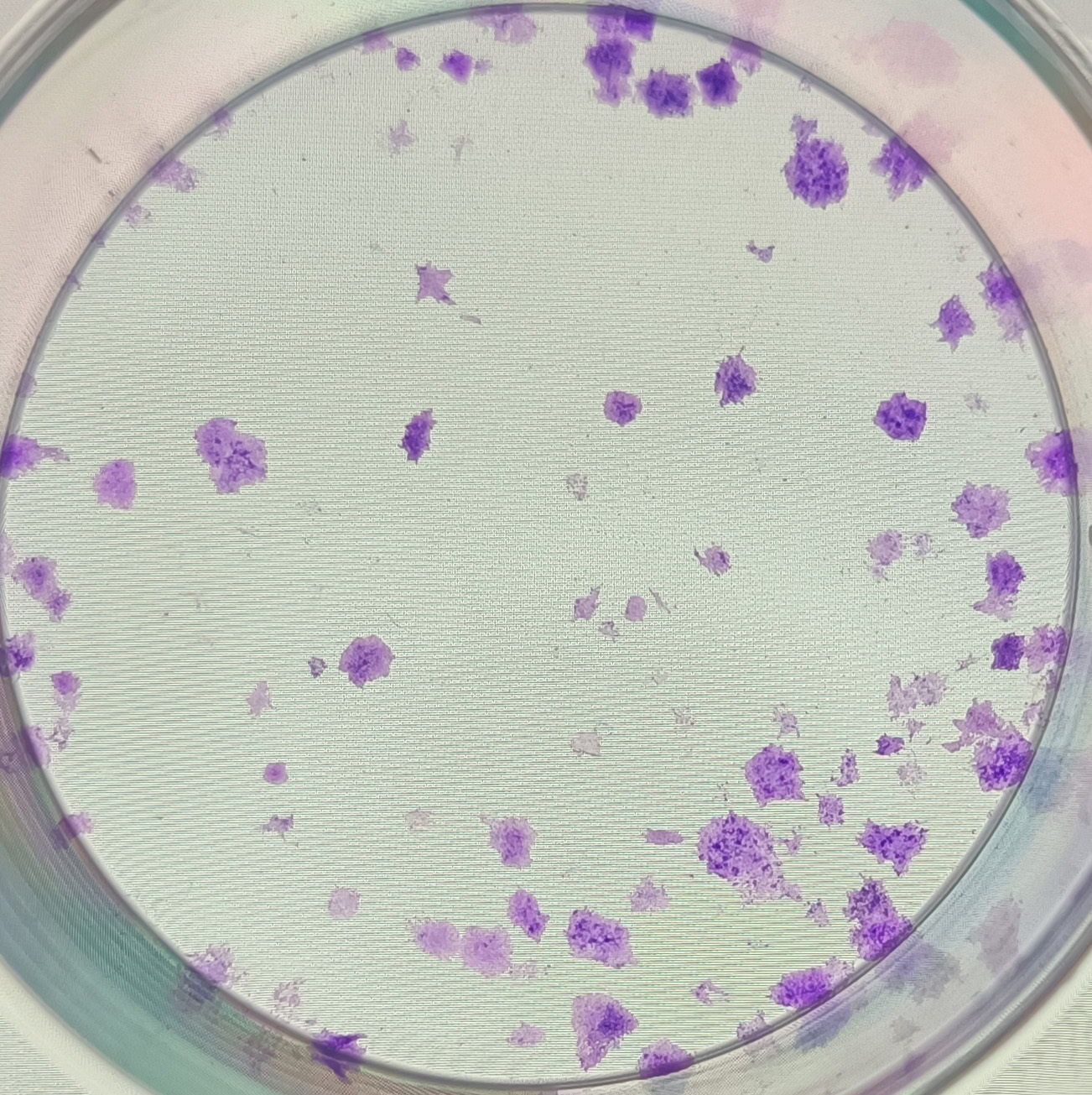

Supplement: Supplemental Information 11 — The clones formed from sample No. 1 in the HOXD11 gene silencing group after 10 days of cell transfection. [file peerj-09-10820-s011.jpg]

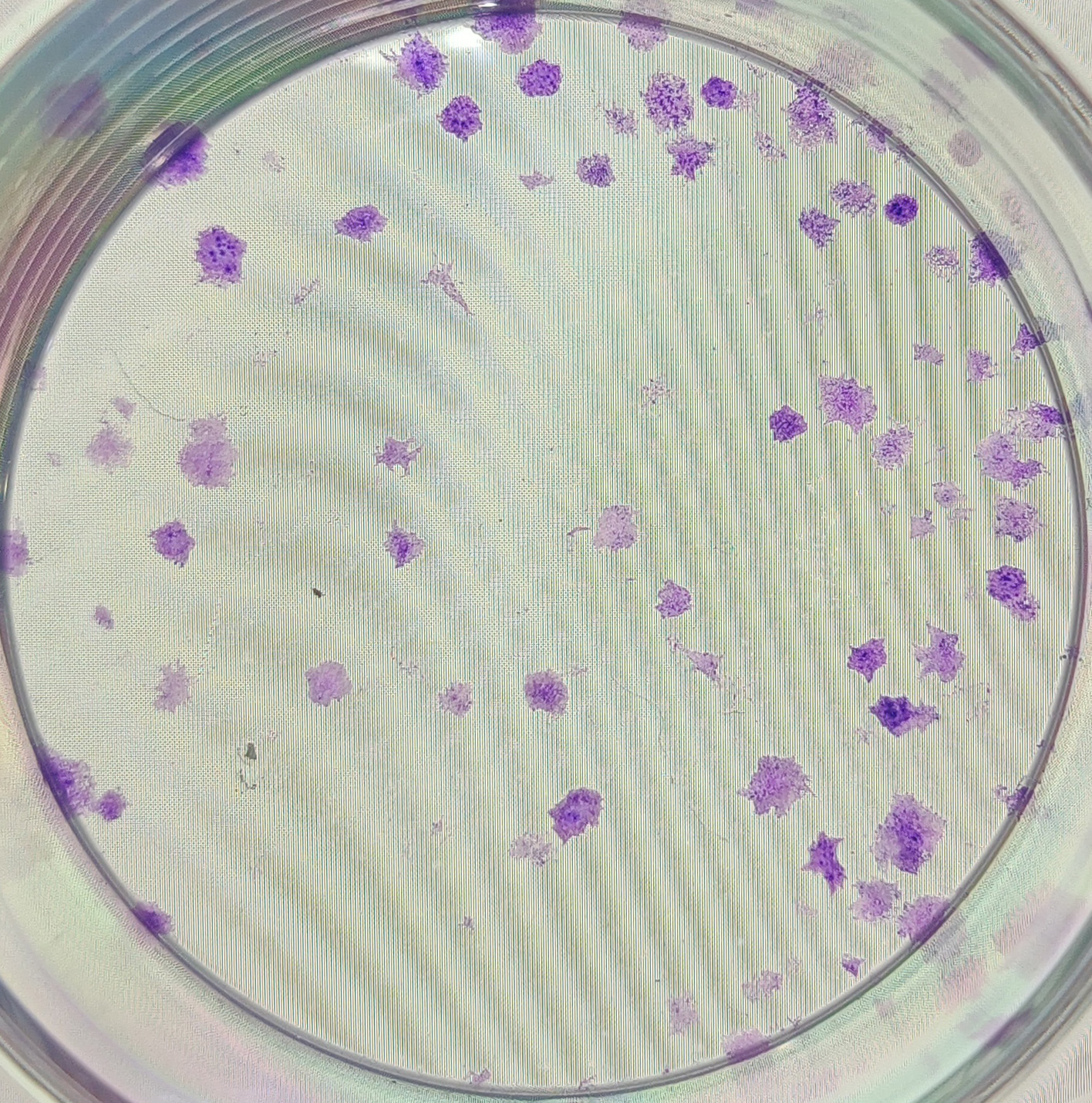

Supplement: Supplemental Information 12 — The clones formed from sample No. 2 in the HOXD11 gene silencing group after 10 days of cell transfection. [file peerj-09-10820-s012.jpg]

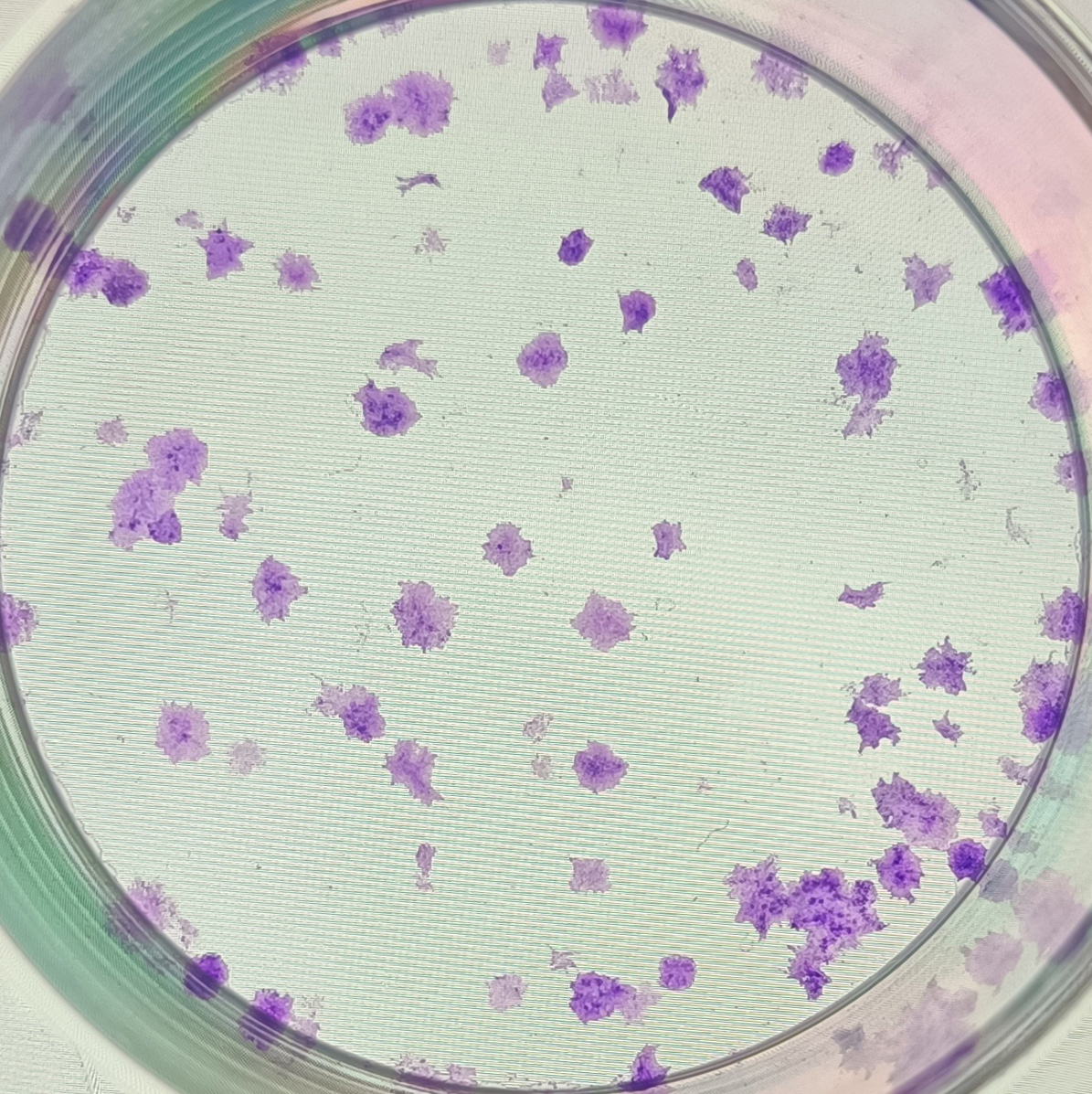

Supplement: Supplemental Information 13 — The clones formed from sample No. 3 in the HOXD11 gene silencing group after 10 days of cell transfection. [file peerj-09-10820-s013.jpg]

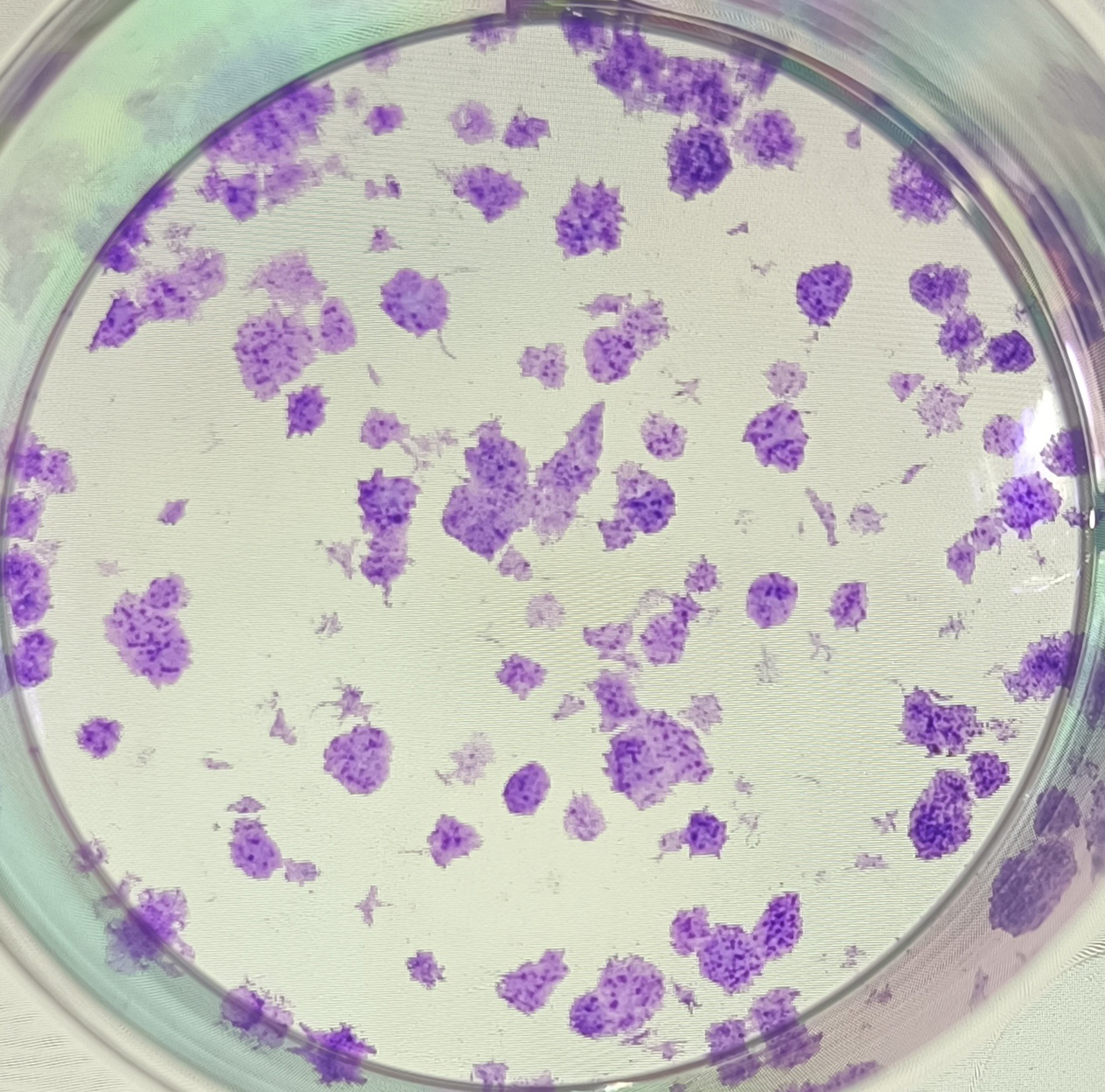

Supplement: Supplemental Information 14 — The clones formed from sample No. 4 in the negative control group after 10 days of cell transfection. [file peerj-09-10820-s014.jpg]

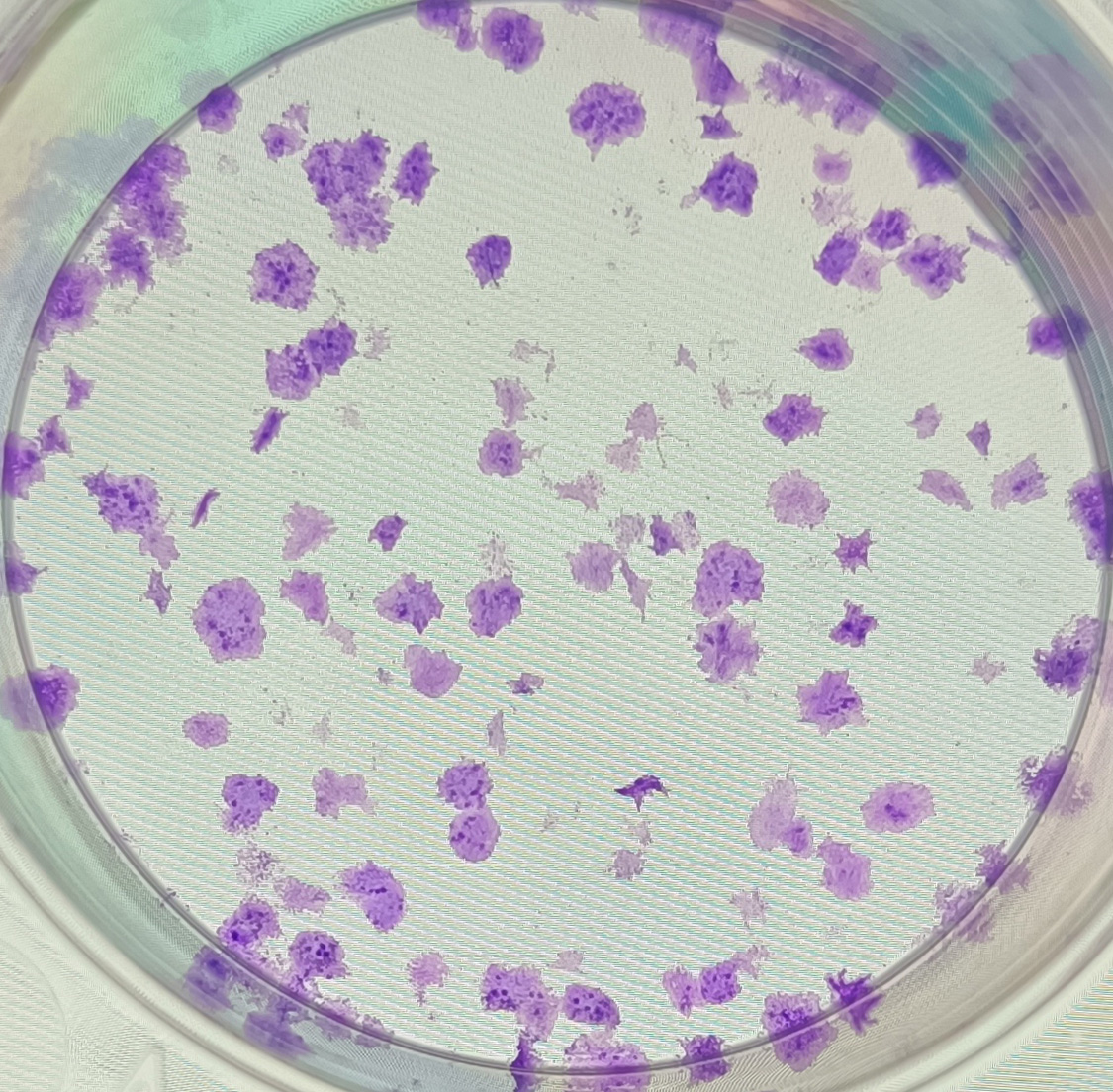

Supplement: Supplemental Information 15 — The clones formed from sample No. 5 in the negative control group after 10 days of cell transfection. [file peerj-09-10820-s015.jpg]

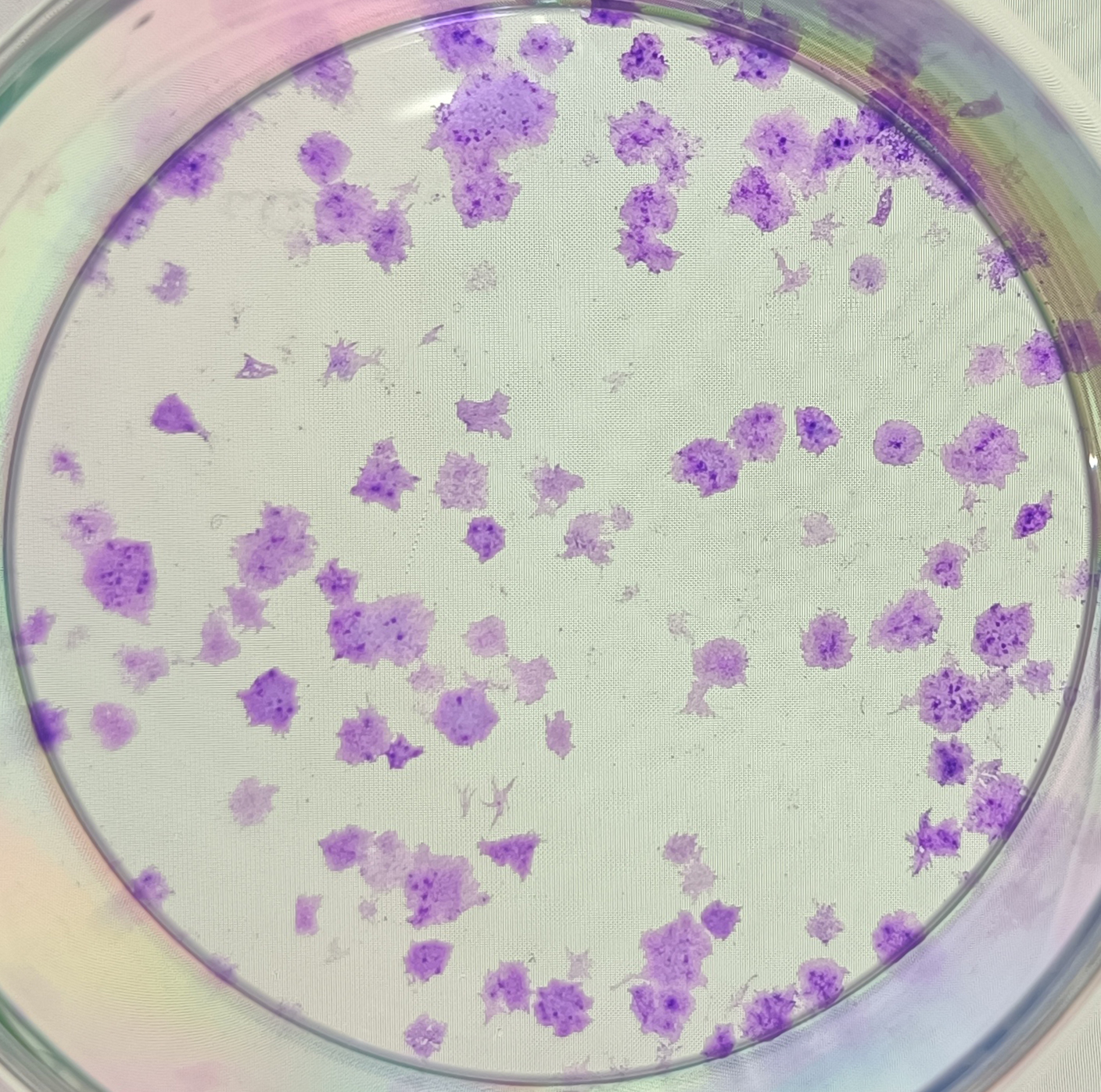

Supplement: Supplemental Information 16 — The clones formed from sample No. 6 in the negative control group after 10 days of cell transfection. [file peerj-09-10820-s016.jpg]

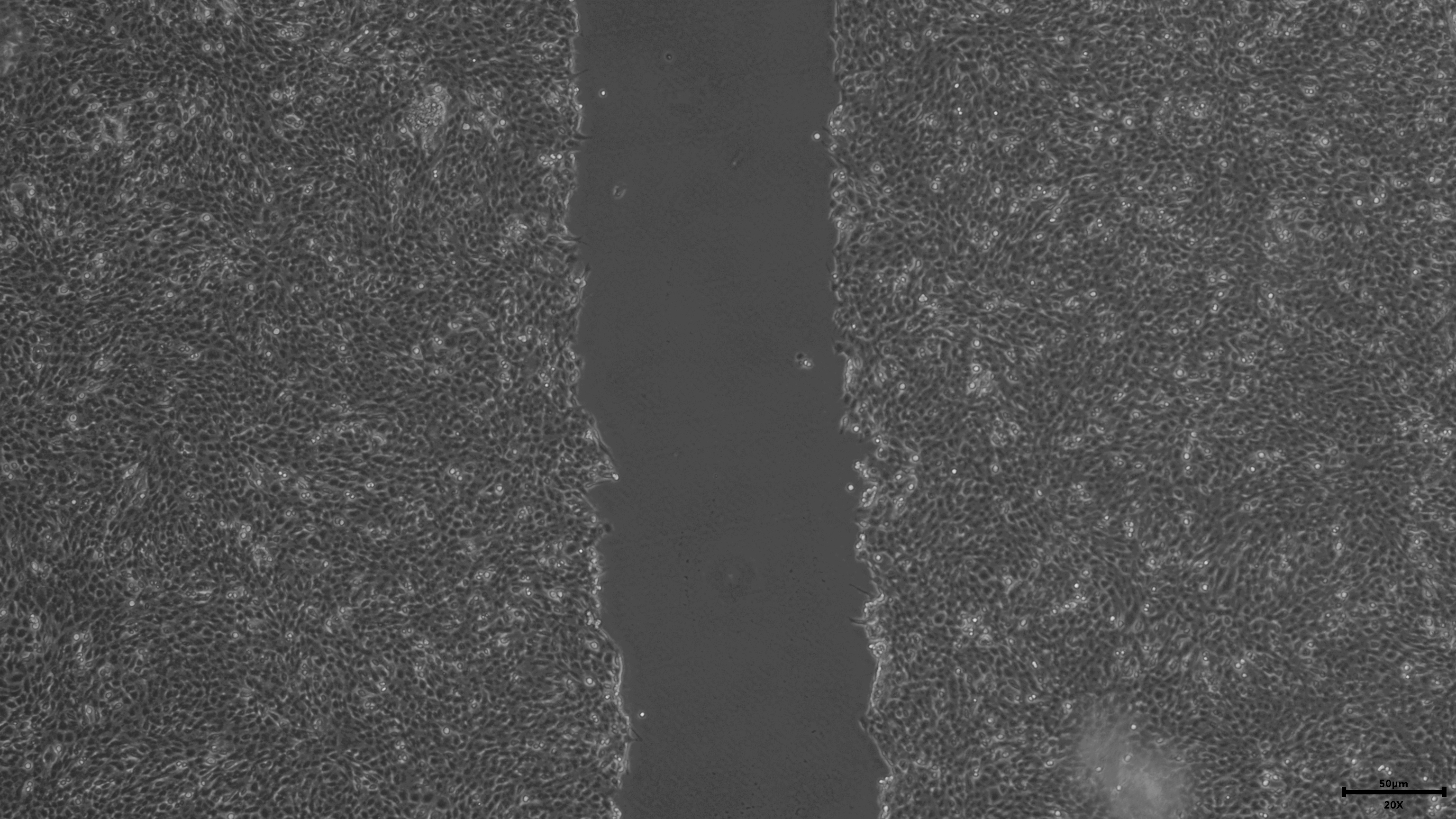

Supplement: Supplemental Information 17 — The scratch of sample No. 1 in the negative control group after 0 h of cell transfection. [file peerj-09-10820-s017.jpg]

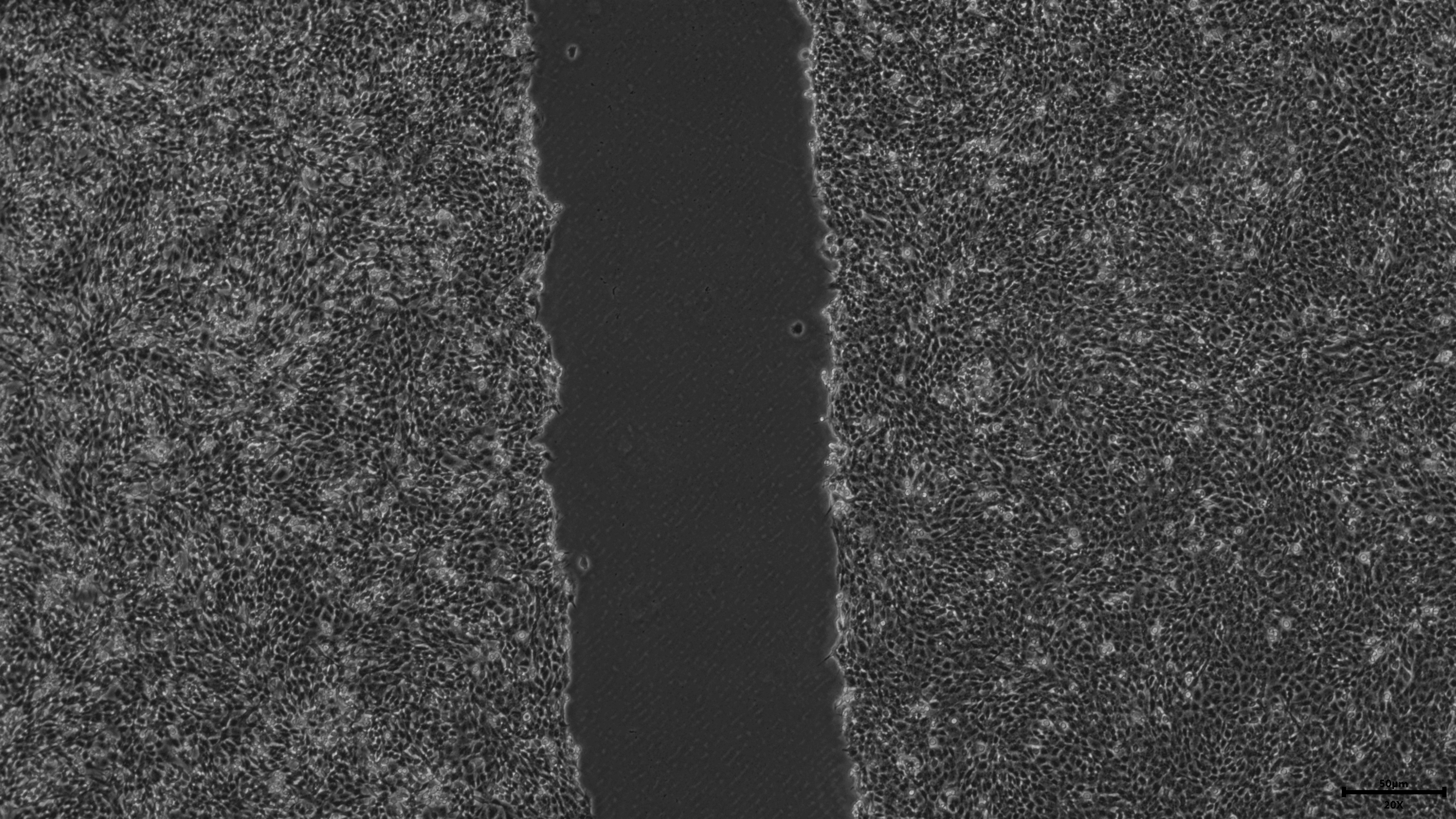

Supplement: Supplemental Information 18 — The scratch of sample No. 2 in the negative control group after 0 h of cell transfection. [file peerj-09-10820-s018.jpg]

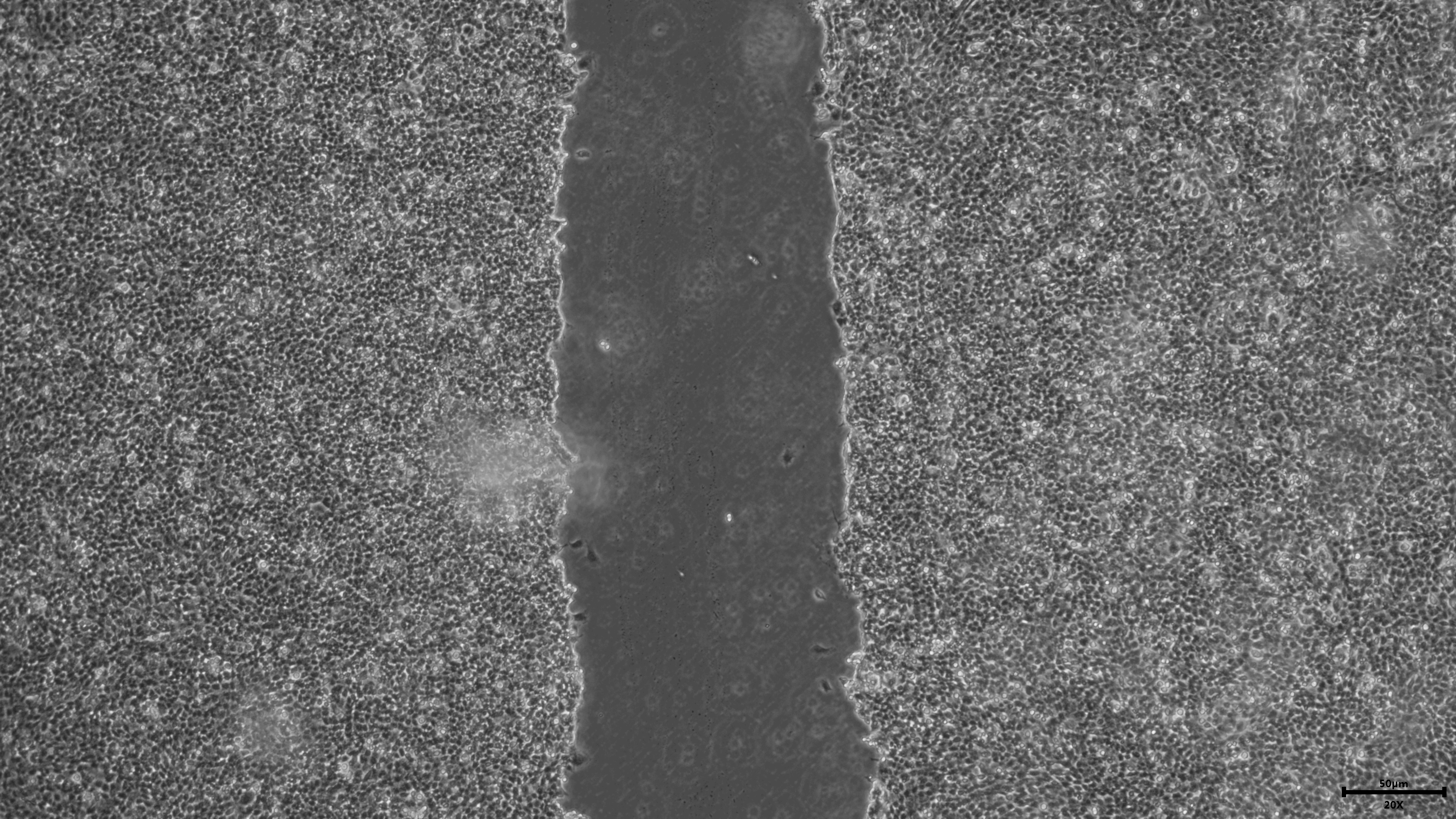

Supplement: Supplemental Information 19 — The scratch of sample No. 3 in the negative control group after 0 h of cell transfection. [file peerj-09-10820-s019.jpg]

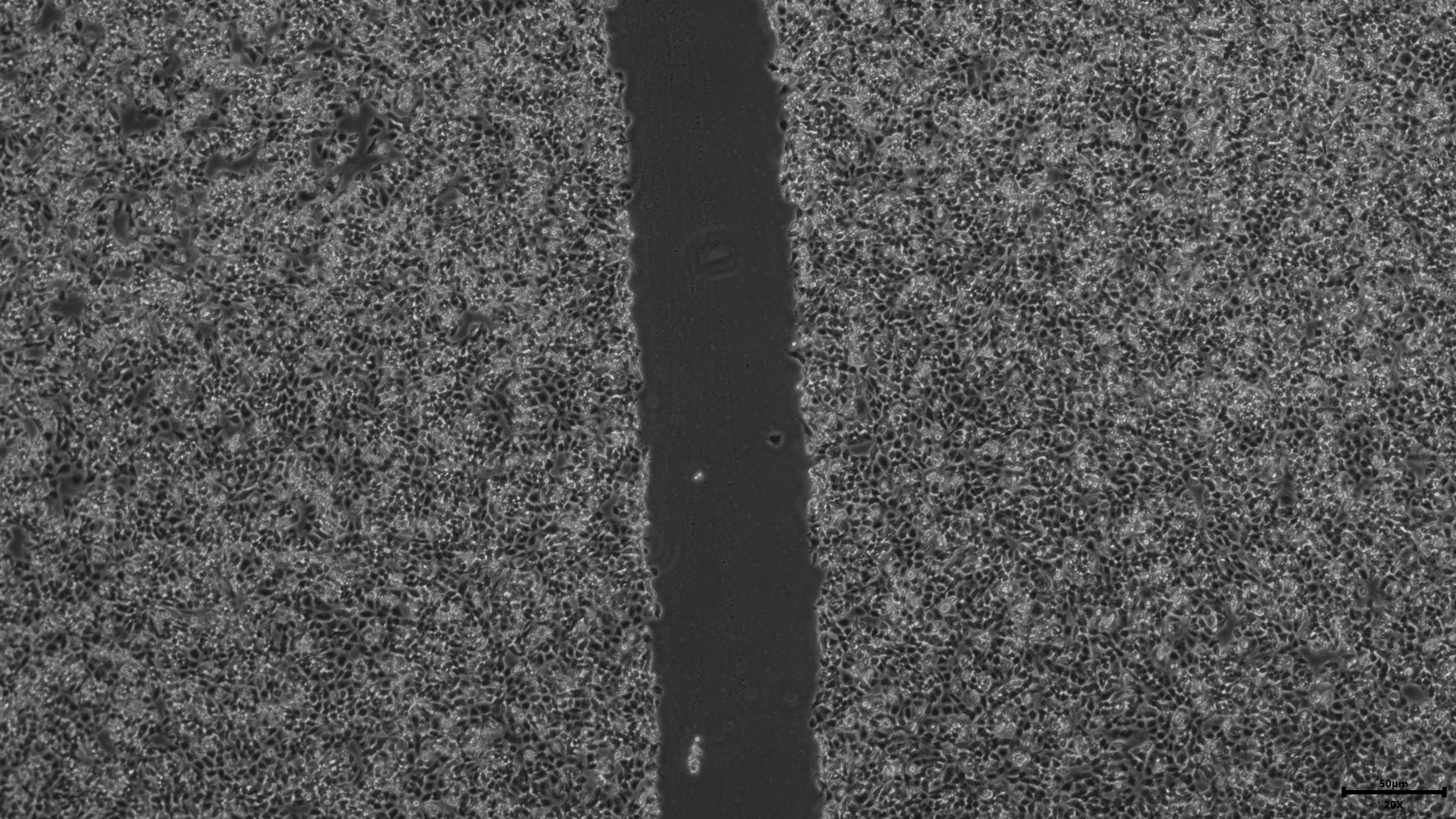

Supplement: Supplemental Information 20 — The scratch of sample No. 1 in the negative control group after 24 h of cell transfection. [file peerj-09-10820-s020.jpg]

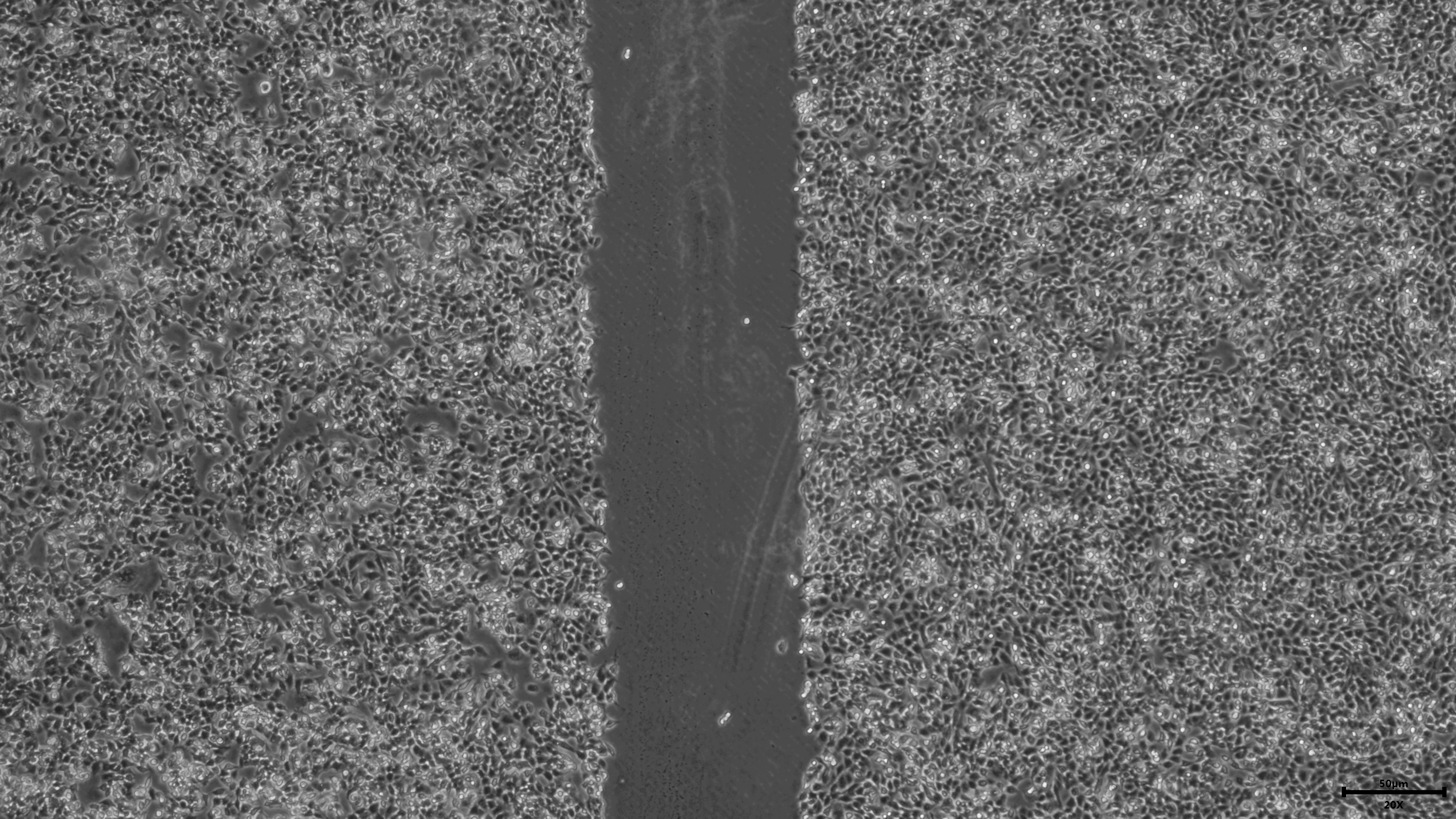

Supplement: Supplemental Information 21 — The scratch of sample No. 2 in the negative control group after 24 h of cell transfection. [file peerj-09-10820-s021.jpg]

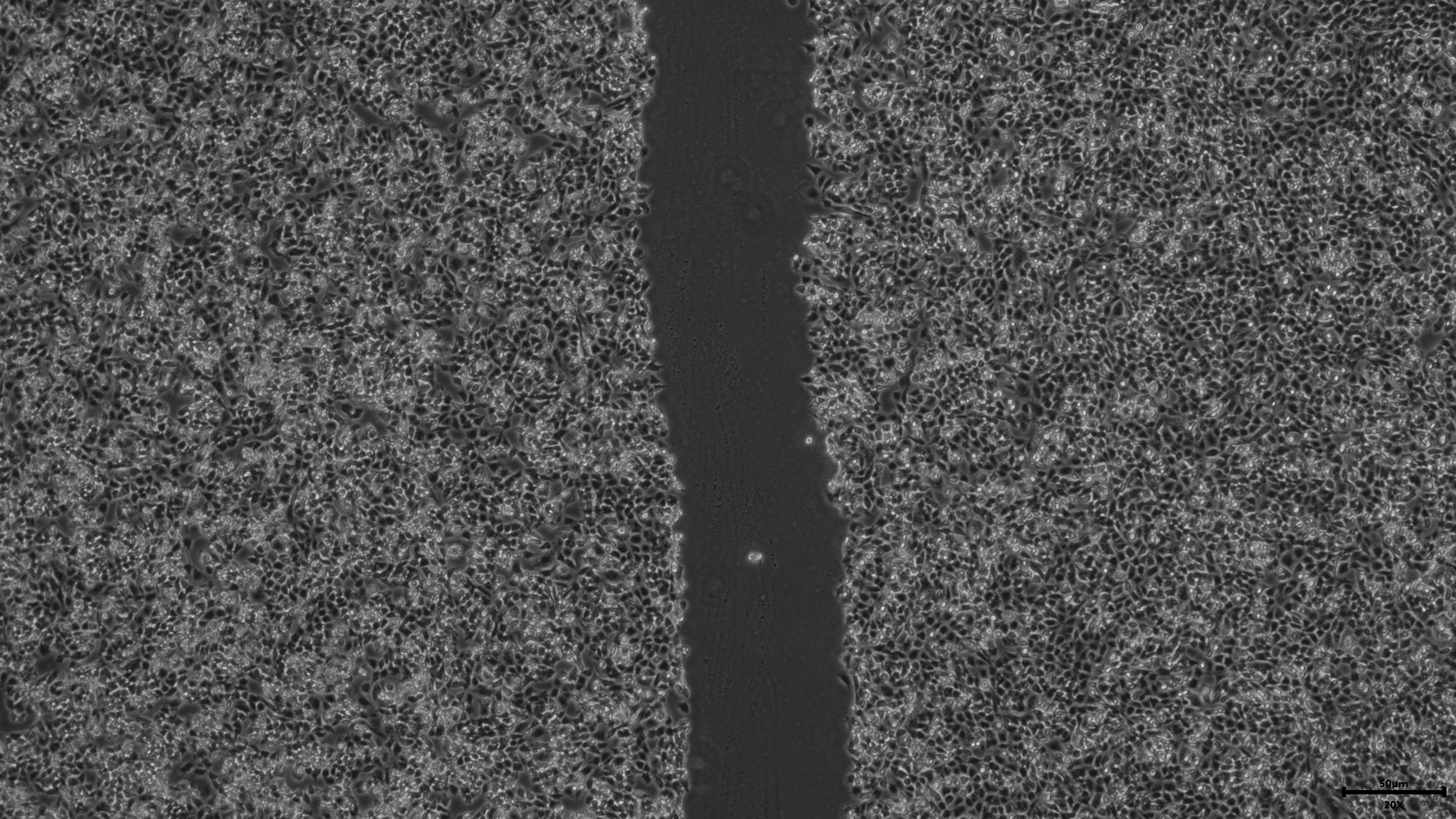

Supplement: Supplemental Information 22 — The scratch of sample No. 3 in the negative control group after 24 h of cell transfection. [file peerj-09-10820-s022.jpg]

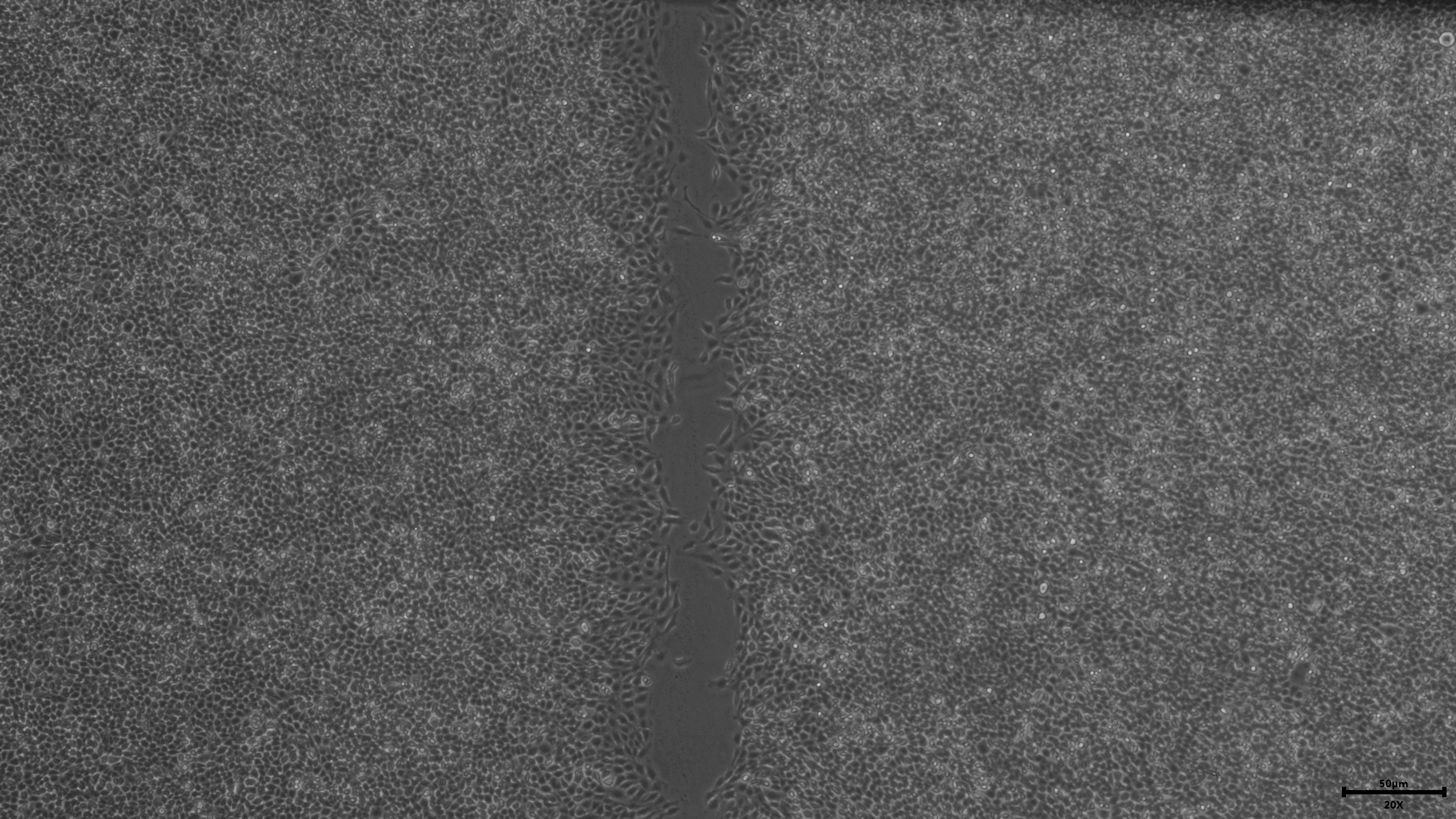

Supplement: Supplemental Information 23 — The scratch of sample No. 1 in the negative control group after 48 h of cell transfection. [file peerj-09-10820-s023.jpg]

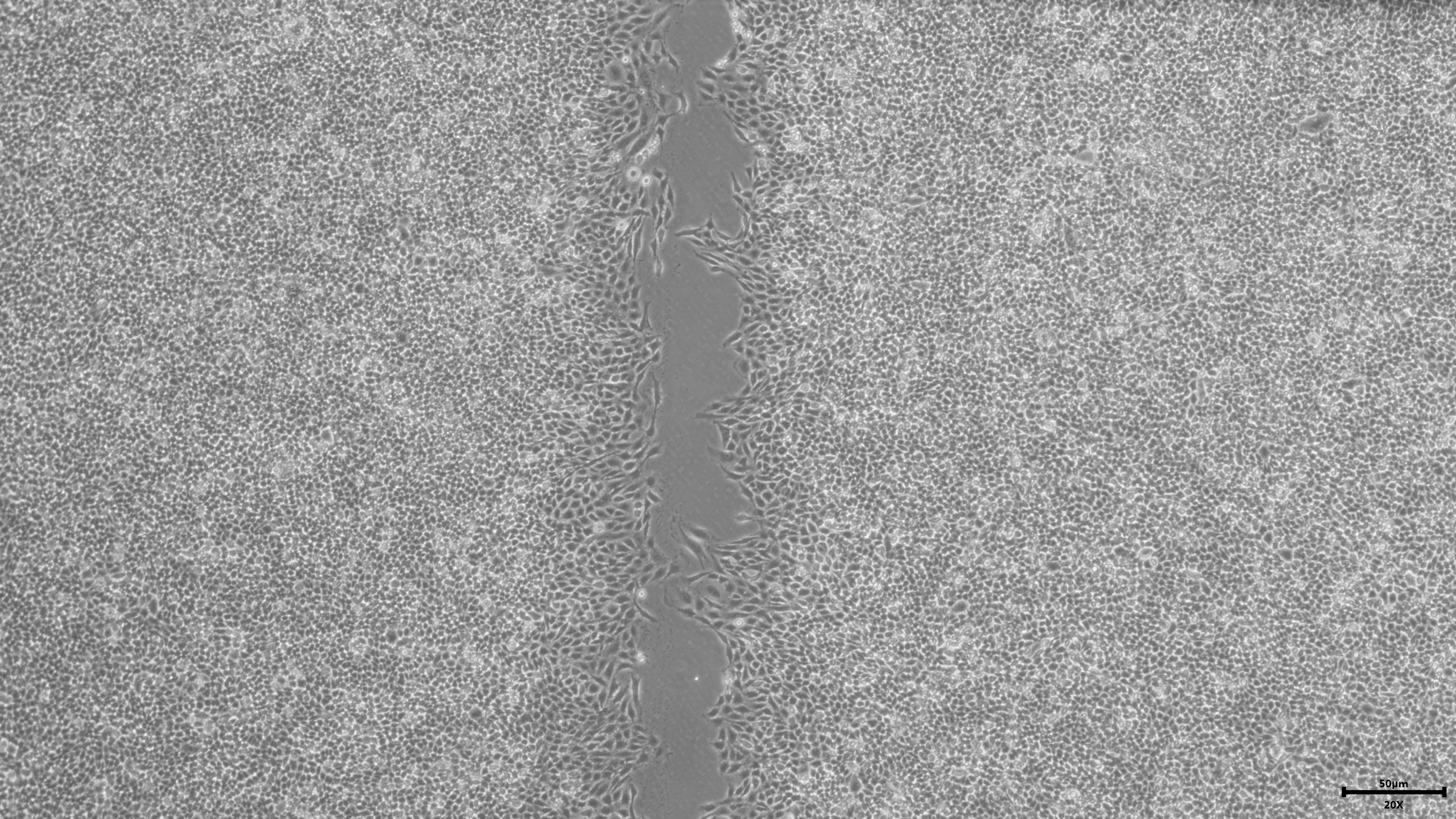

Supplement: Supplemental Information 24 — The scratch of sample No. 2 in the negative control group after 48 h of cell transfection. [file peerj-09-10820-s024.jpg]

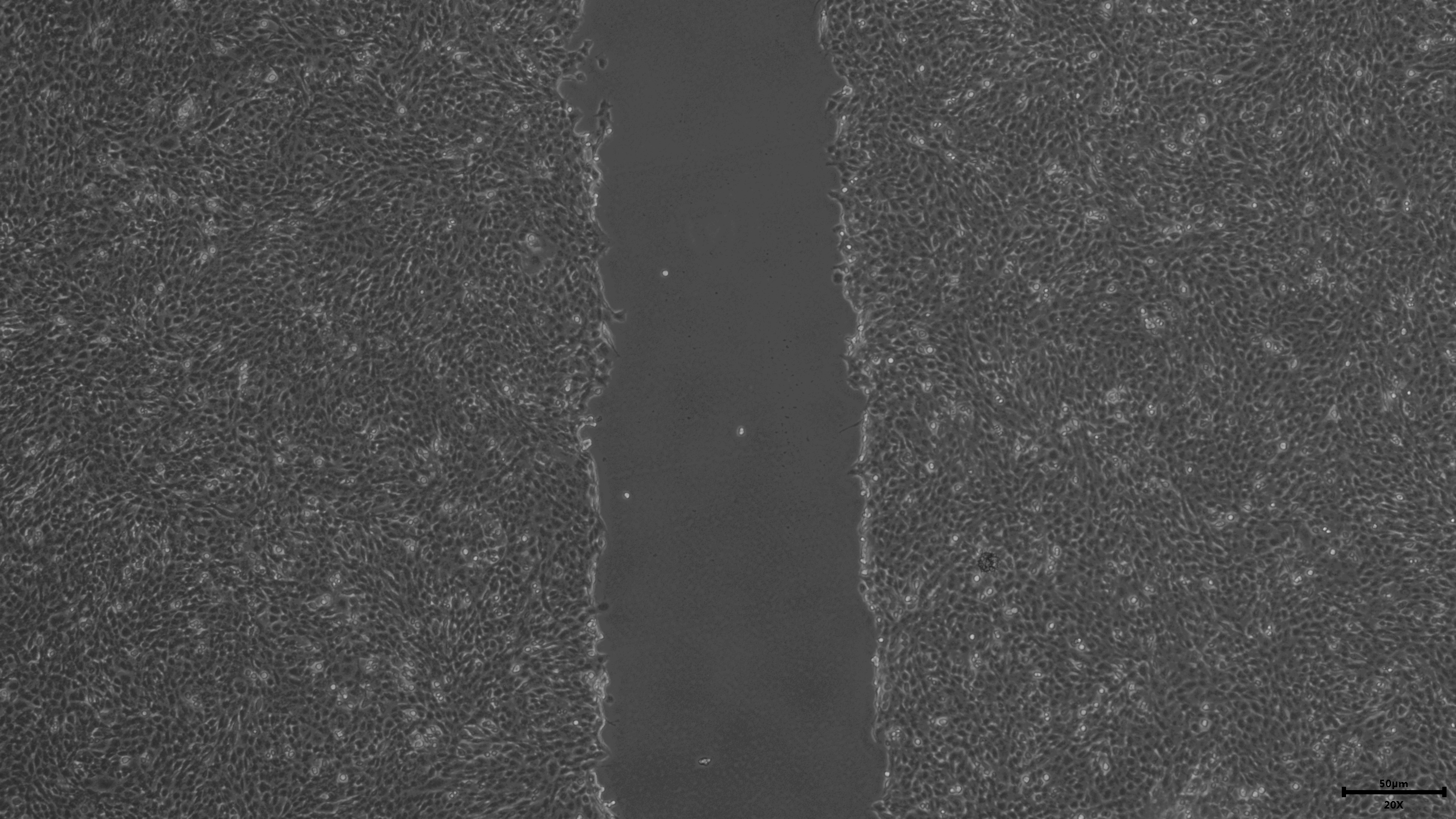

Supplement: Supplemental Information 25 — The scratch of sample No. 4 in the HOXD11 gene silencing group after 0 h of cell transfection. [file peerj-09-10820-s025.jpg]

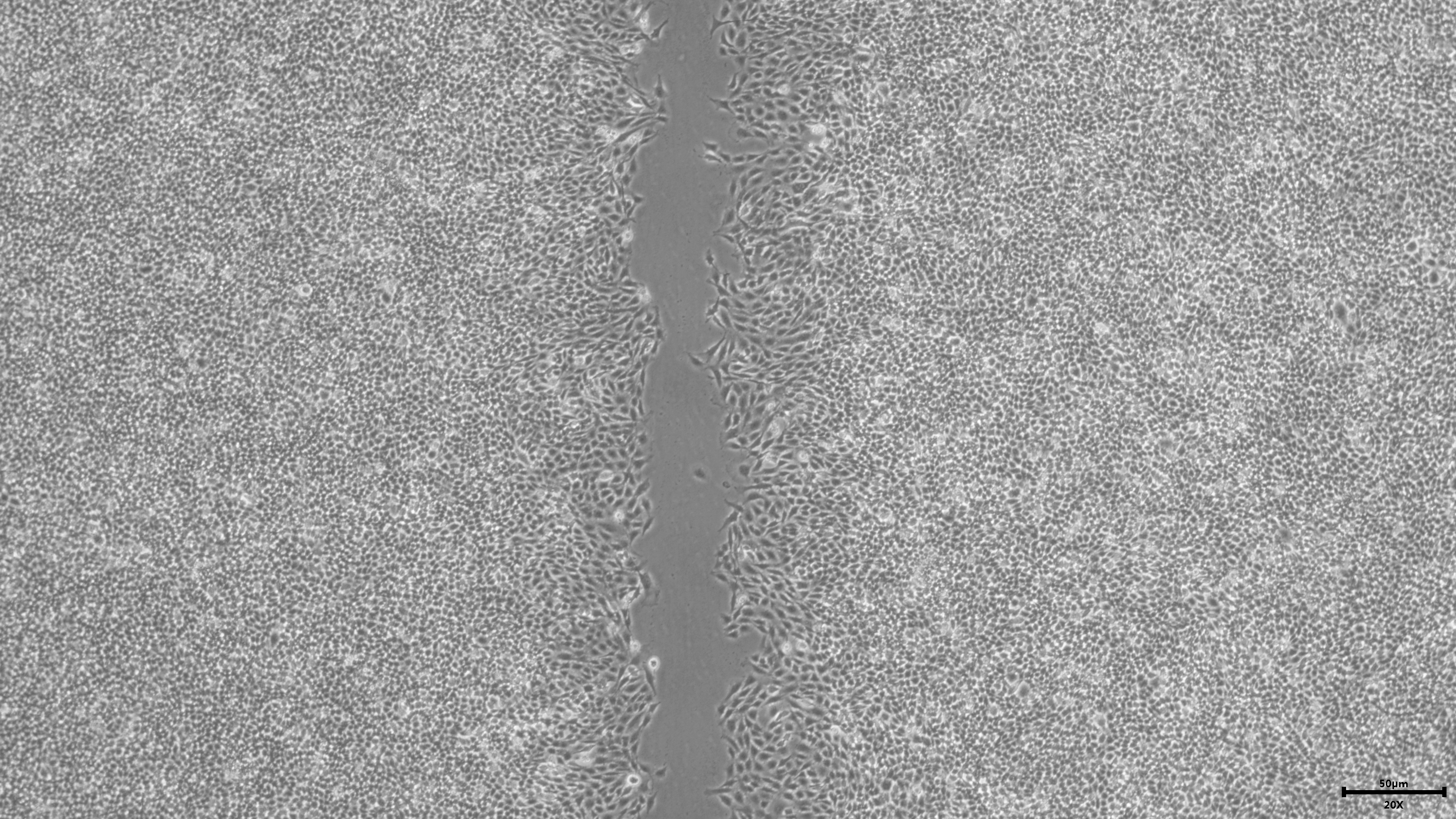

Supplement: Supplemental Information 26 — The scratch of sample No. 3 in the negative control group after 48 h of cell transfection. [file peerj-09-10820-s026.jpg]

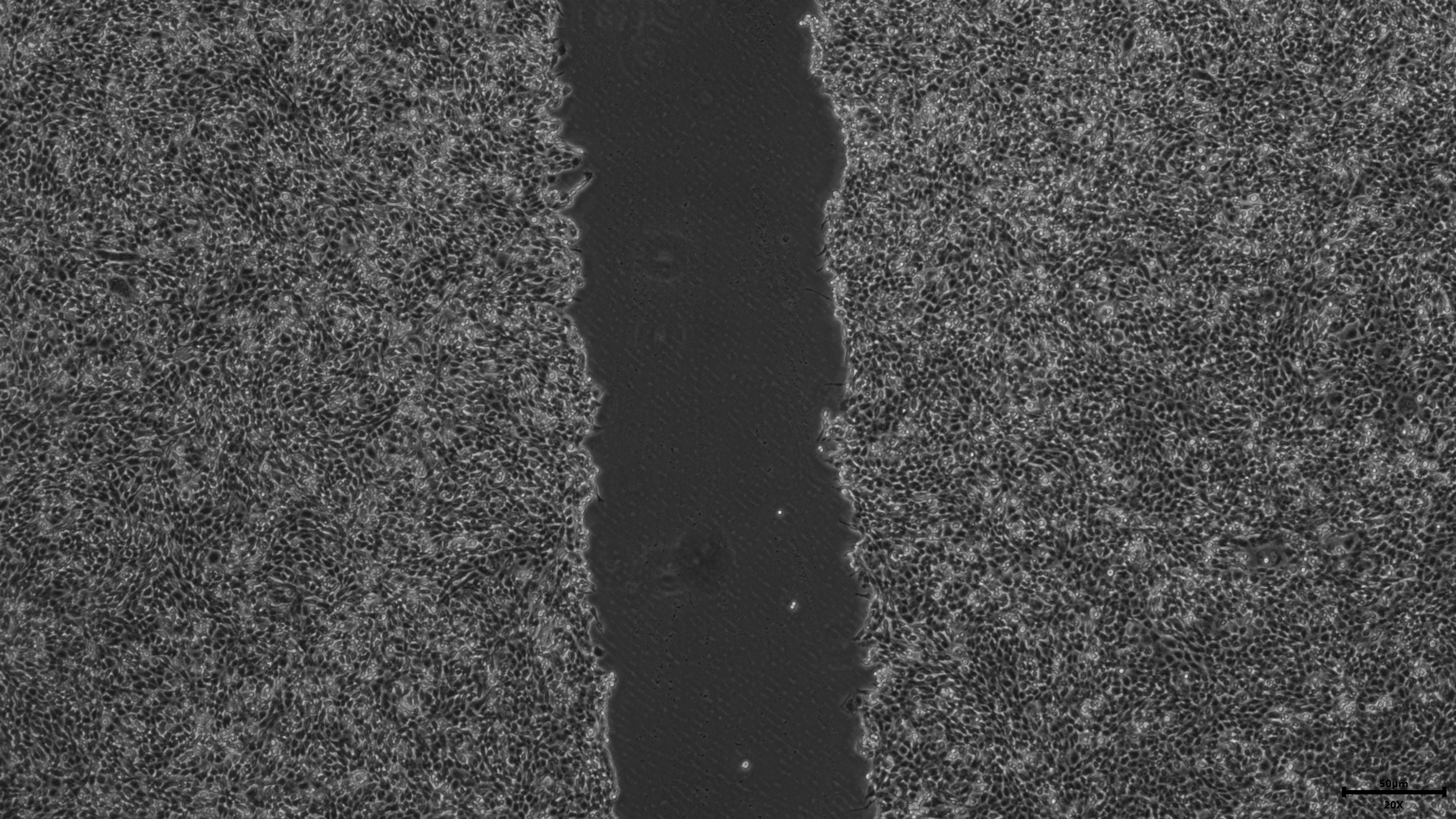

Supplement: Supplemental Information 27 — The scratch of sample No. 5 in the HOXD11 gene silencing group after 0 h of cell transfection. [file peerj-09-10820-s027.jpg]

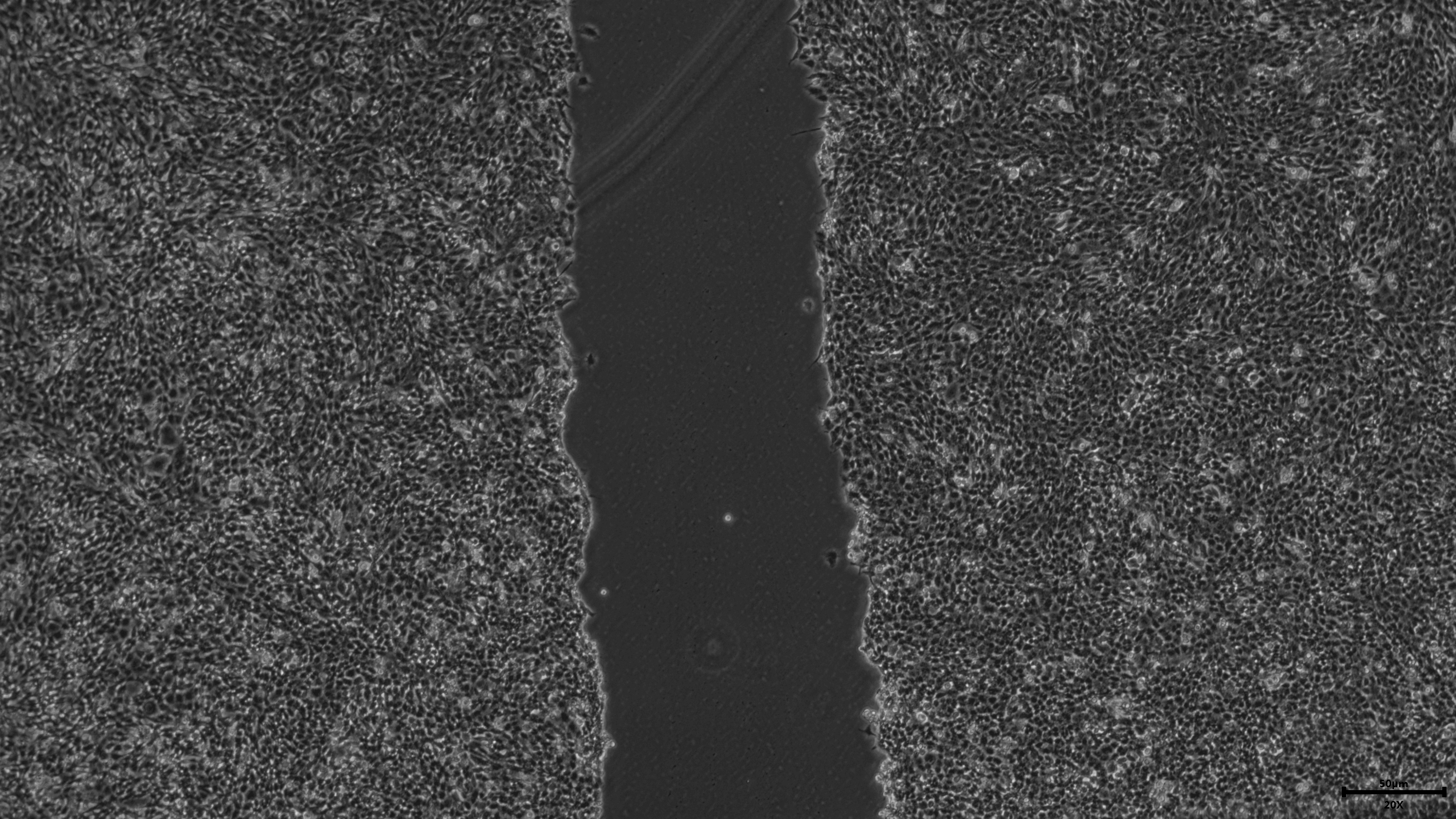

Supplement: Supplemental Information 28 — The scratch of sample No. 6 in the HOXD11 gene silencing group after 0 h of cell transfection. [file peerj-09-10820-s028.jpg]

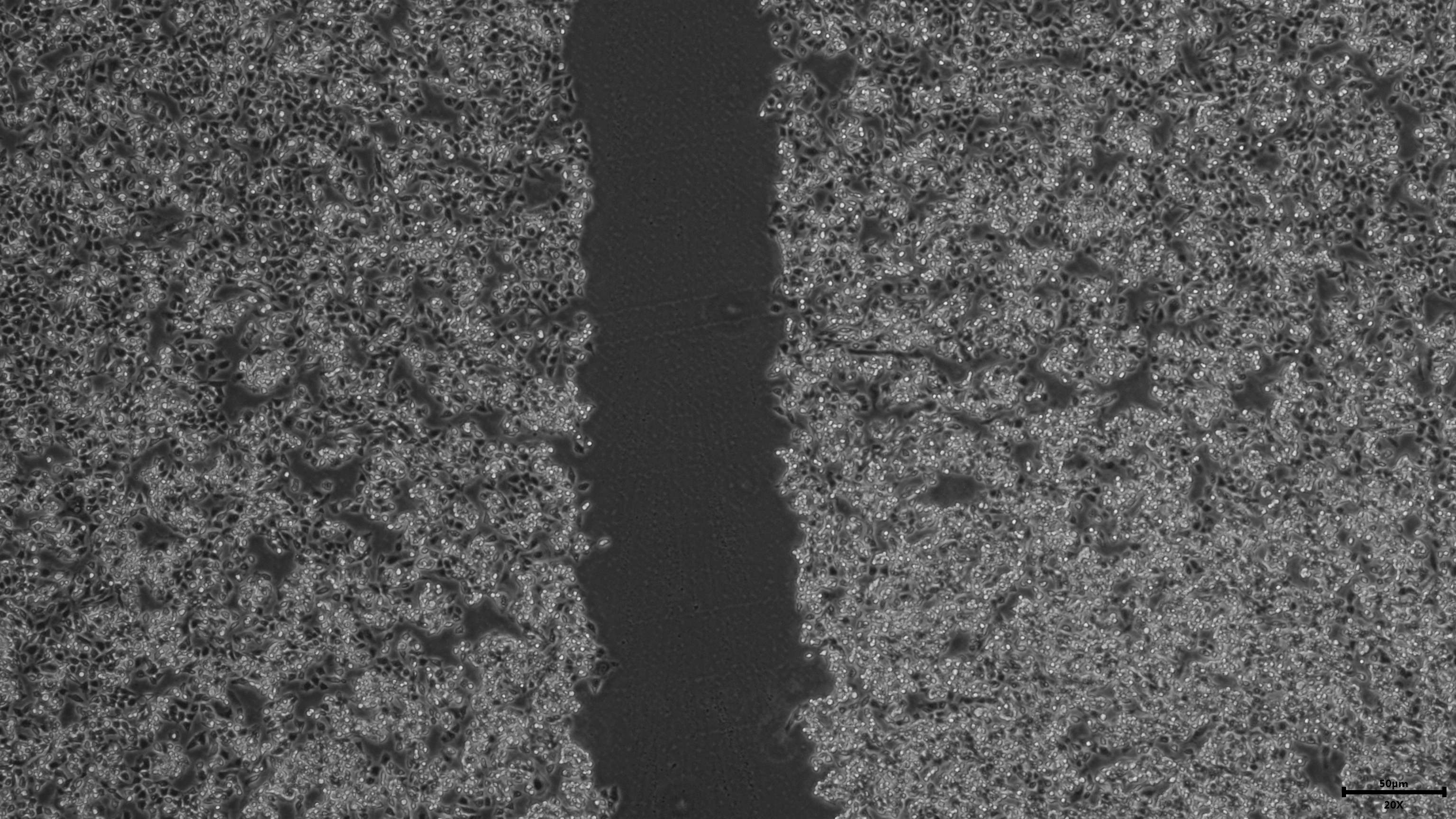

Supplement: Supplemental Information 29 — The scratch of sample No. 4 in the HOXD11 gene silencing group after 24 h of cell transfection. [file peerj-09-10820-s029.jpg]

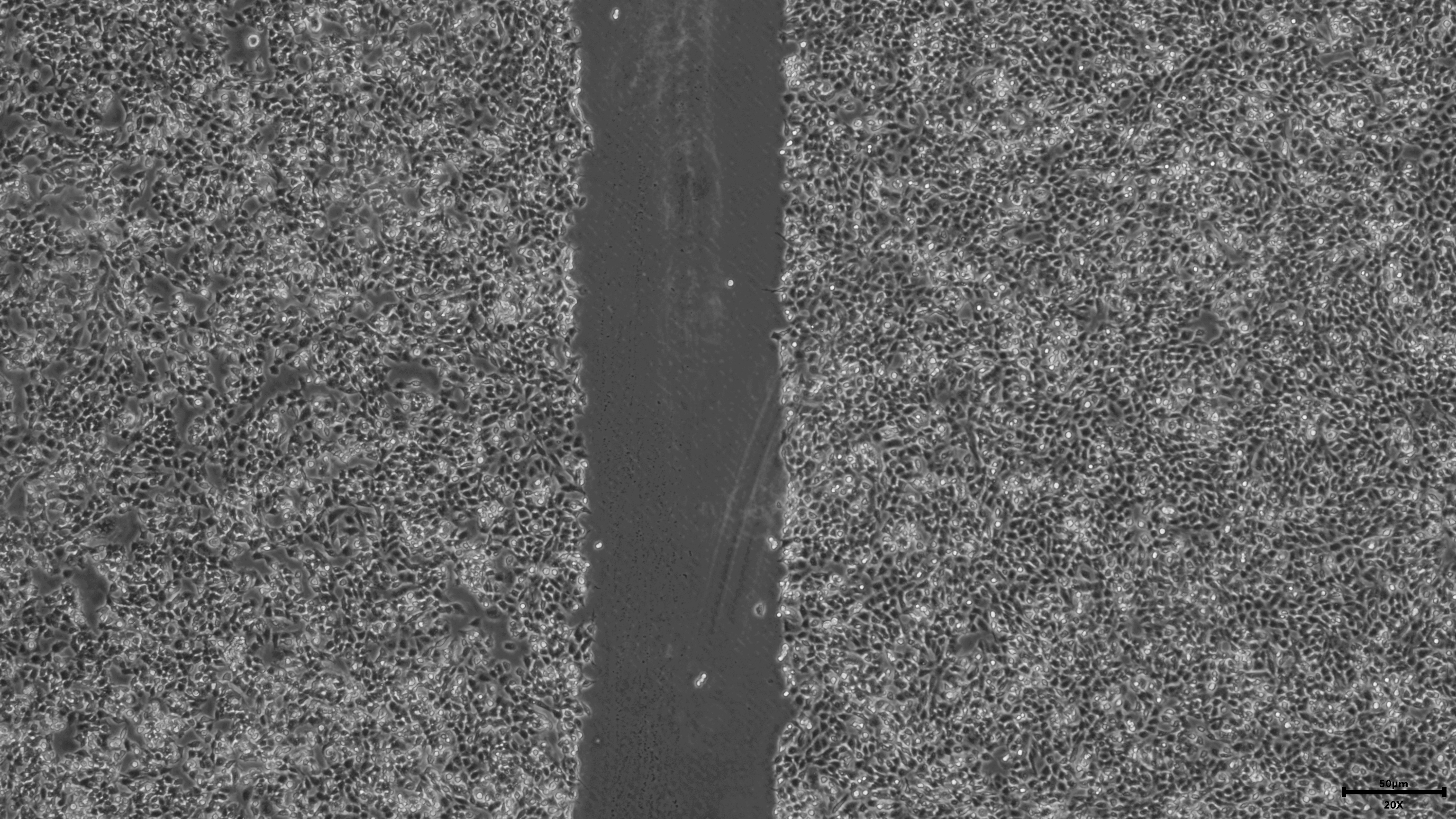

Supplement: Supplemental Information 30 — The scratch of sample No. 5 in the HOXD11 gene silencing group after 24 h of cell transfection. [file peerj-09-10820-s030.jpg]

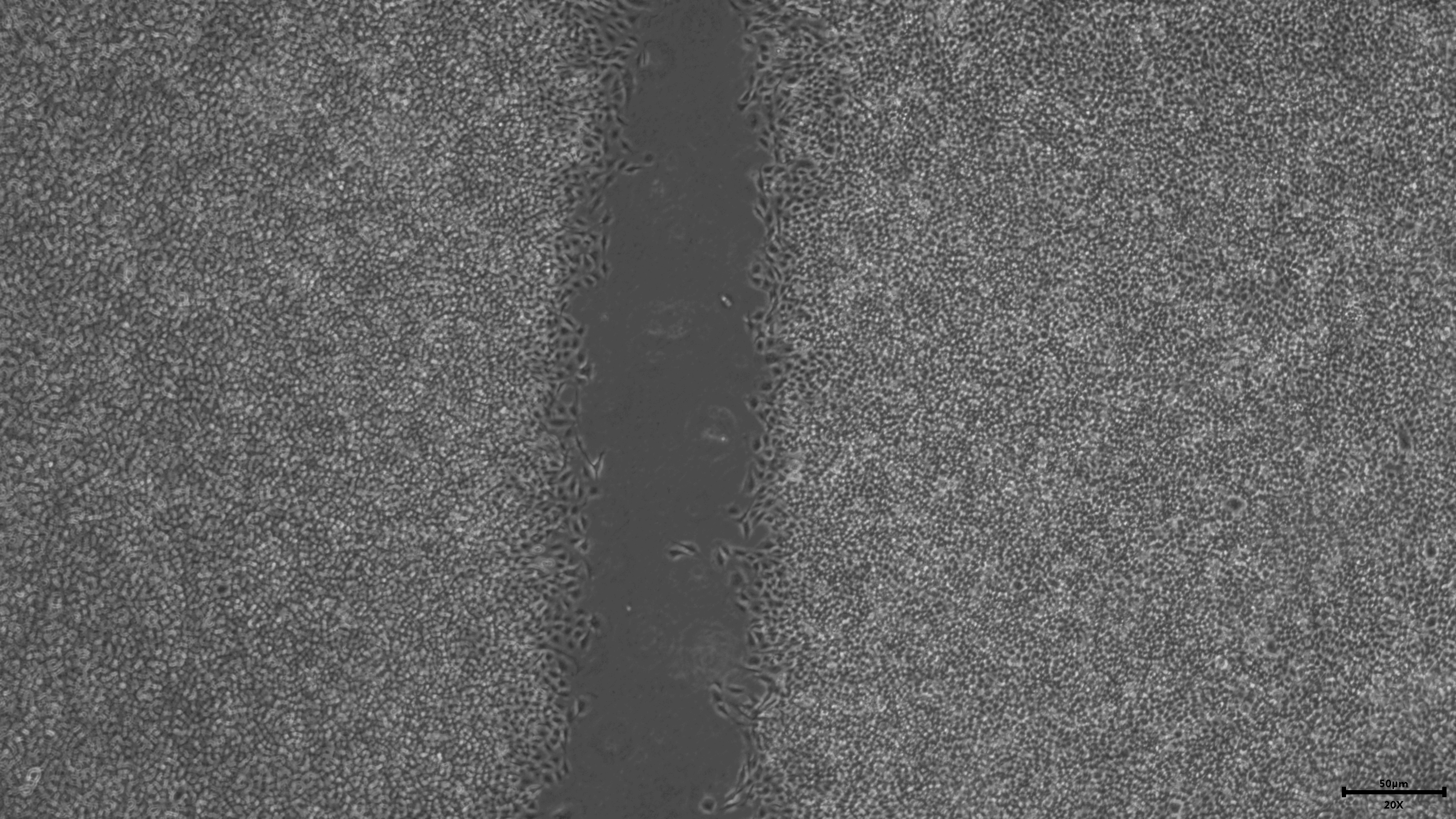

Supplement: Supplemental Information 31 — The scratch of sample No. 4 in the HOXD11 gene silencing group after 48 h of cell transfection. [file peerj-09-10820-s031.jpg]

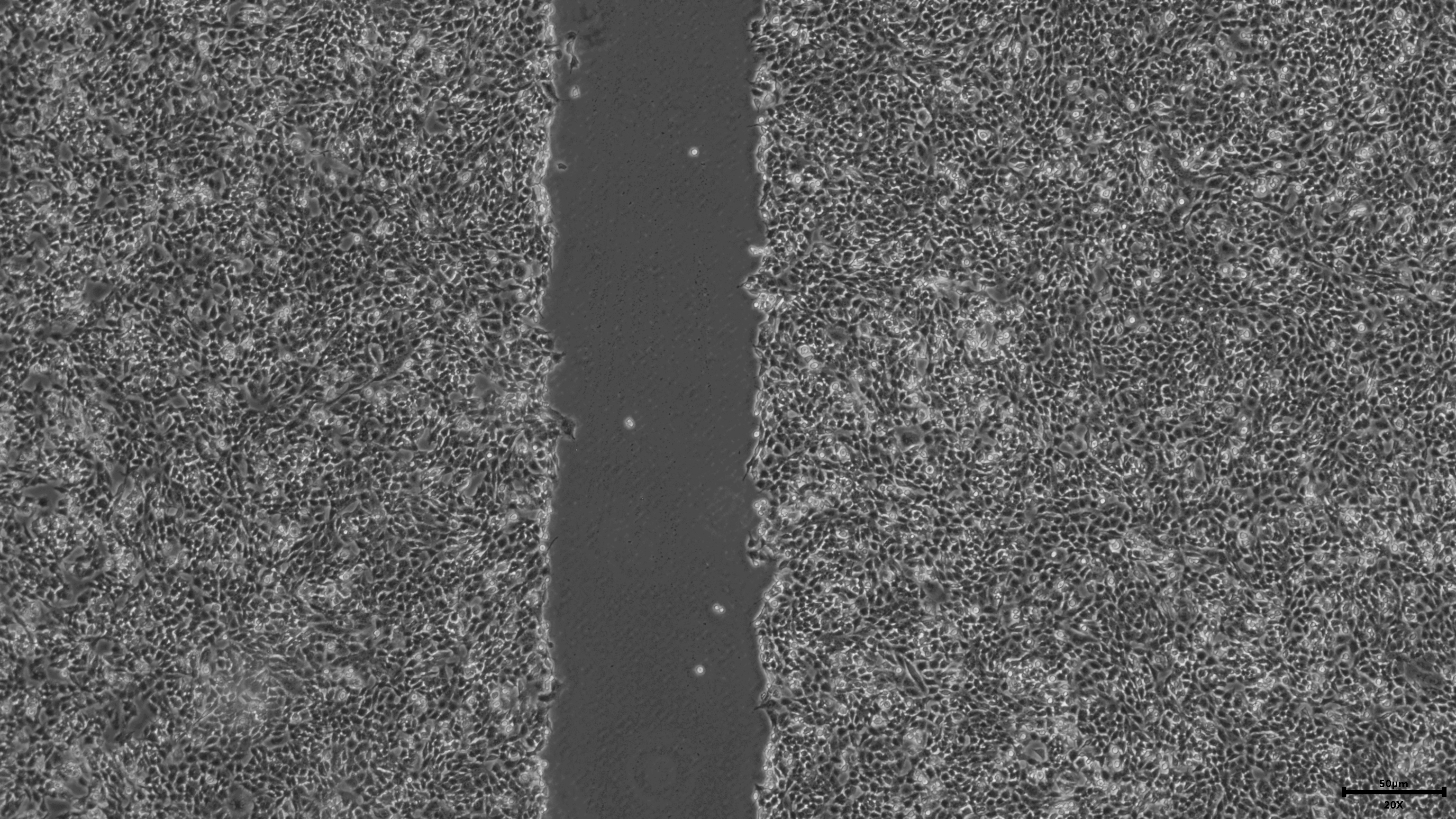

Supplement: Supplemental Information 32 — The scratch of sample No. 6 in the HOXD11 gene silencing group after 24 h of cell transfection. [file peerj-09-10820-s032.jpg]

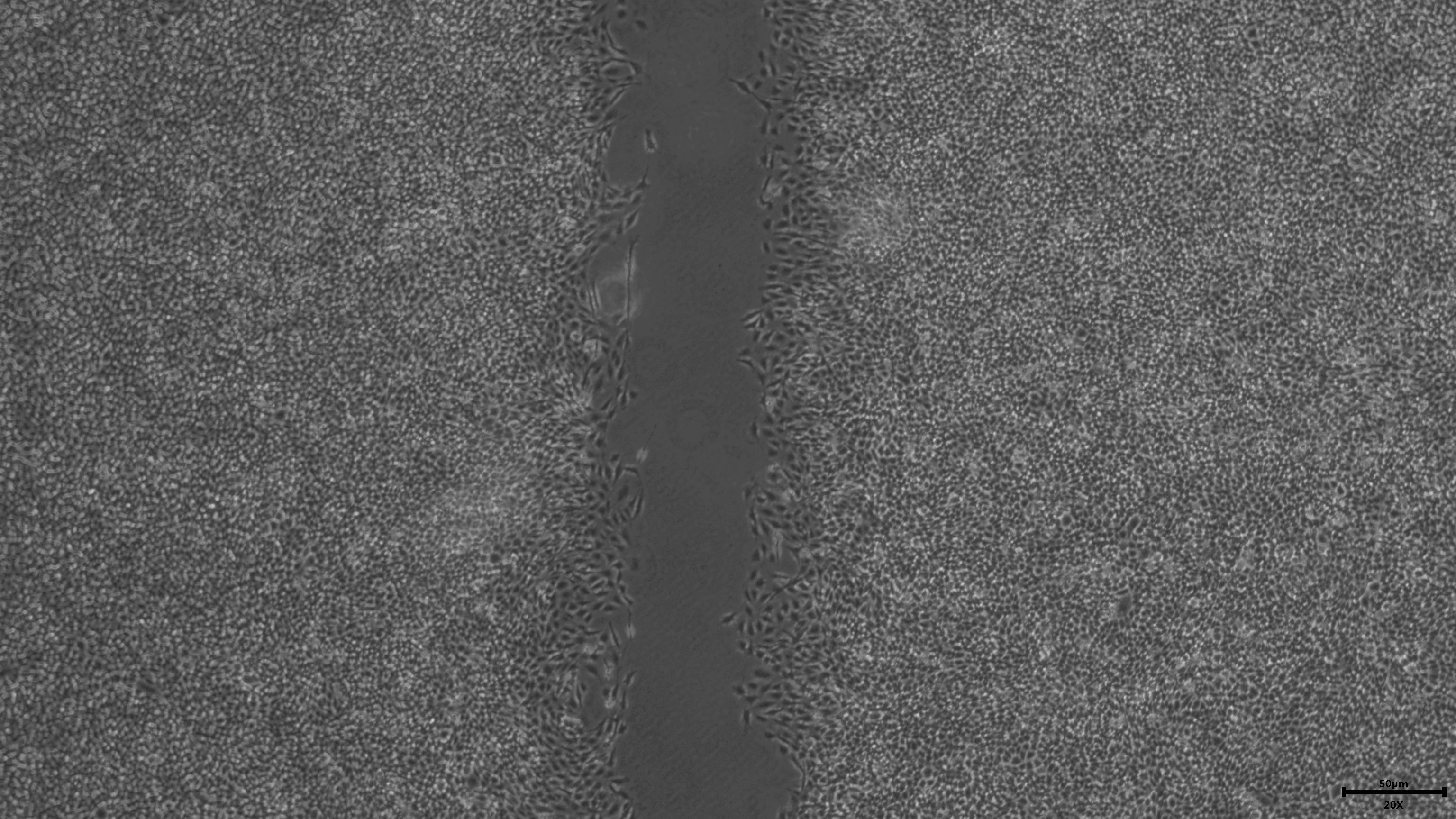

Supplement: Supplemental Information 33 — The scratch of sample No. 5 in the HOXD11 gene silencing group after 48 h of cell transfection. [file peerj-09-10820-s033.jpg]

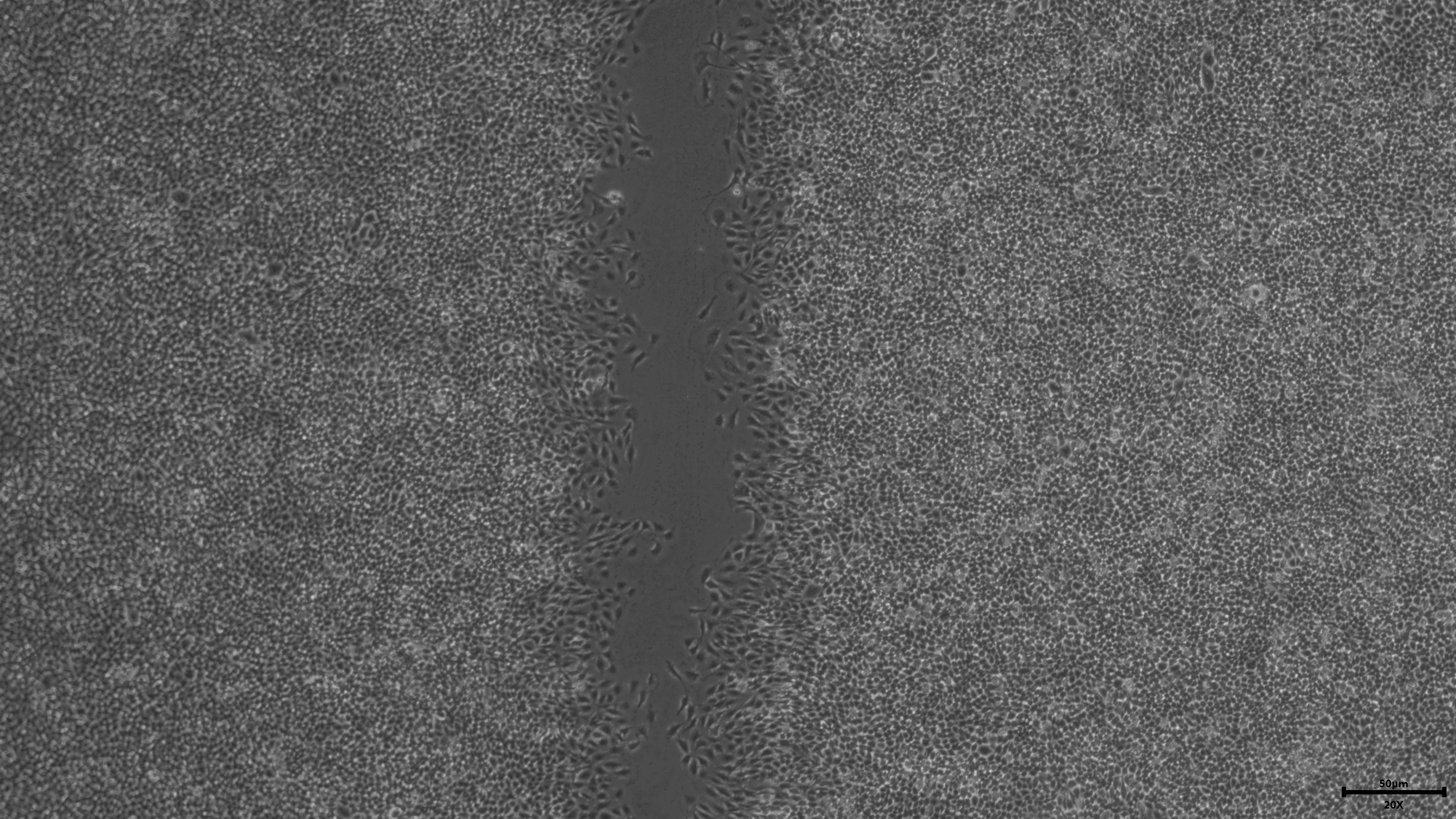

Supplement: Supplemental Information 34 — The scratch of sample No. 6 in the HOXD11 gene silencing group after 48 h of cell transfection. [file peerj-09-10820-s034.jpg]

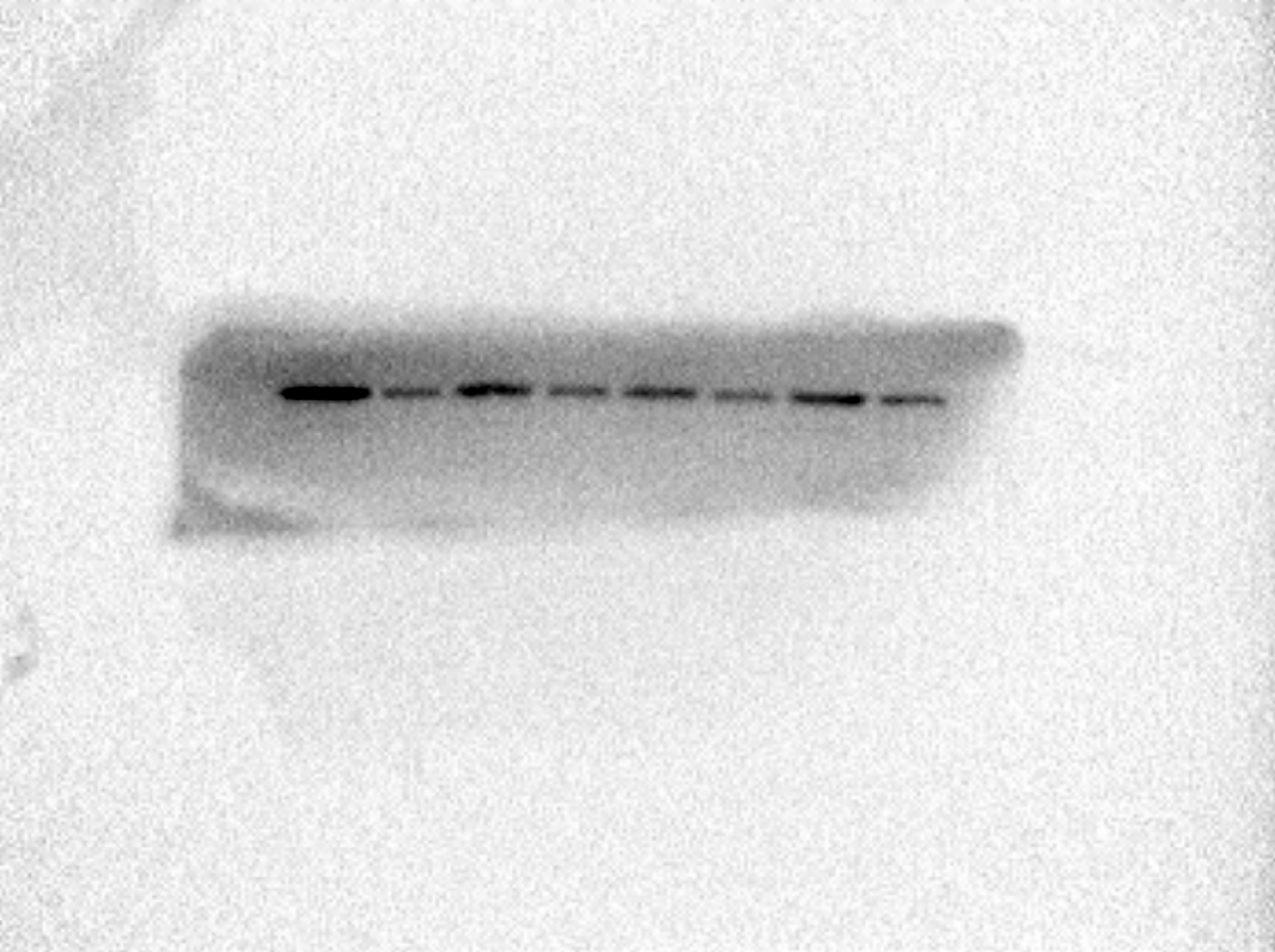

Supplement: Supplemental Information 43 — The expression of the key protein Cdc25c in the G2 phase of the cell cycle by western blotting after cell transfection. [file peerj-09-10820-s043.jpg]

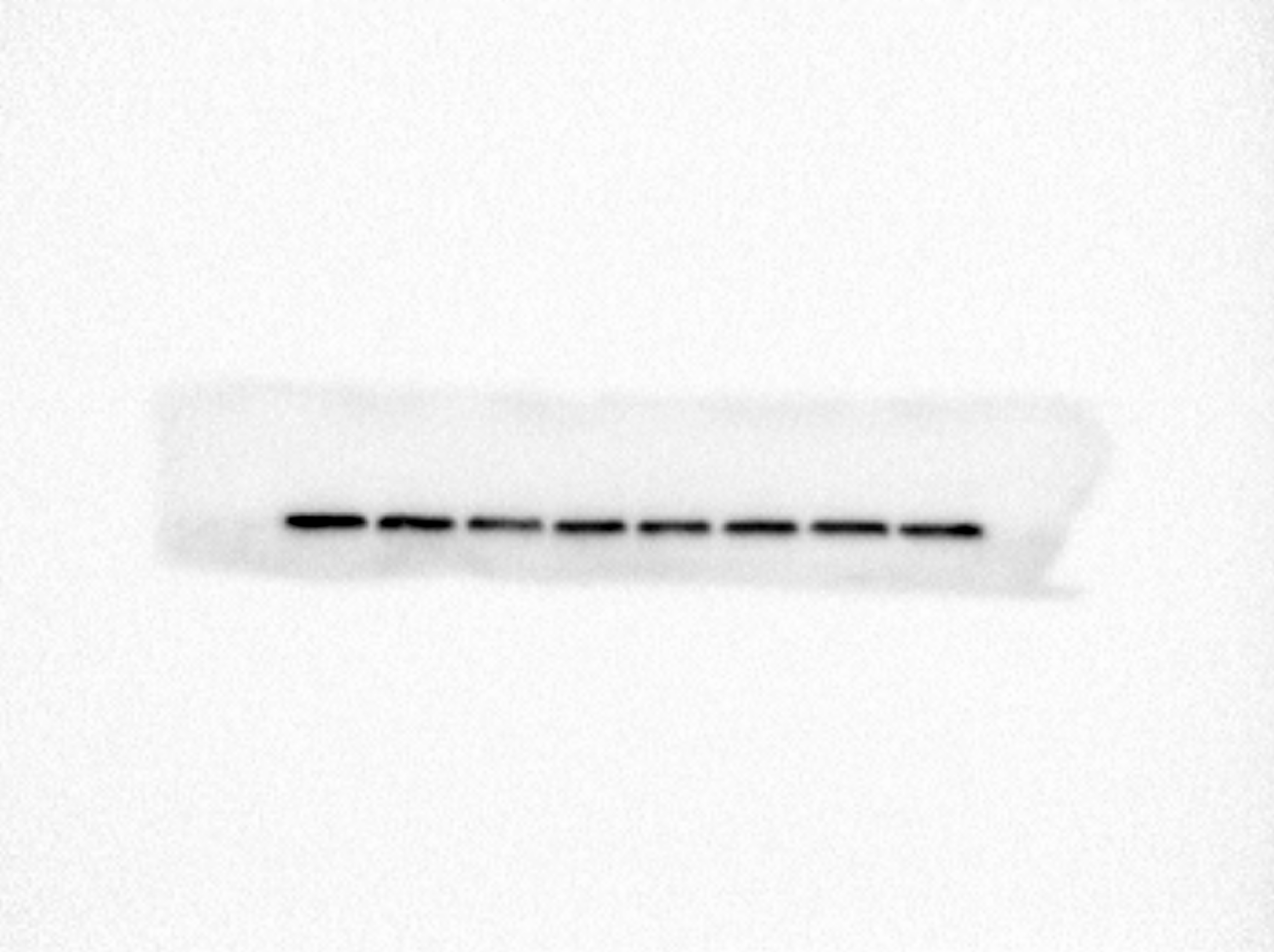

Supplement: Supplemental Information 44 — The expression of the internal reference protein GAPDH by western blotting after cell transfection. [file peerj-09-10820-s044.jpg]

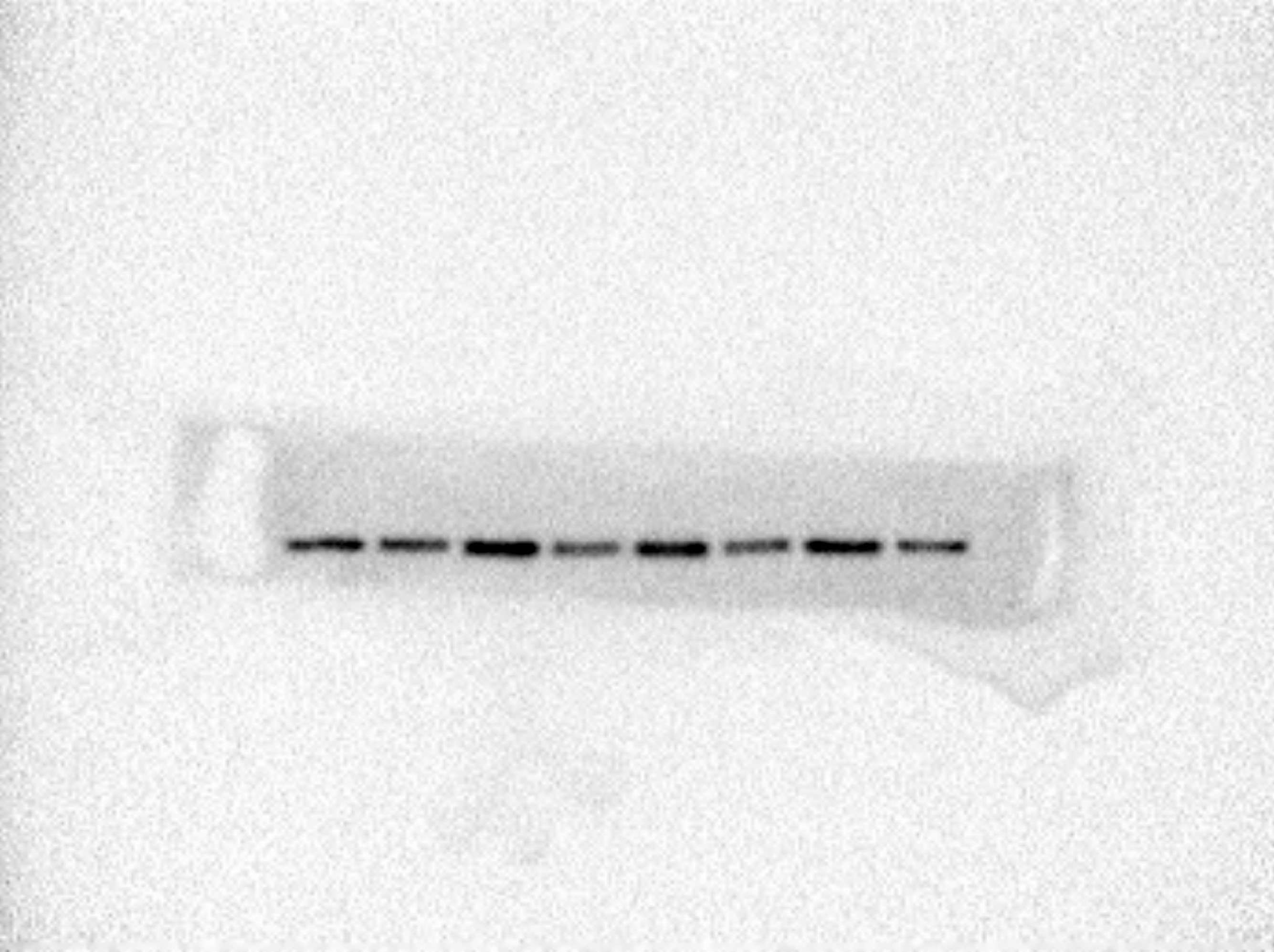

Supplement: Supplemental Information 45 — The expression of the key protein CDK1 in the G2 phase of the cell cycle by western blotting after cell transfection. [file peerj-09-10820-s045.jpg]

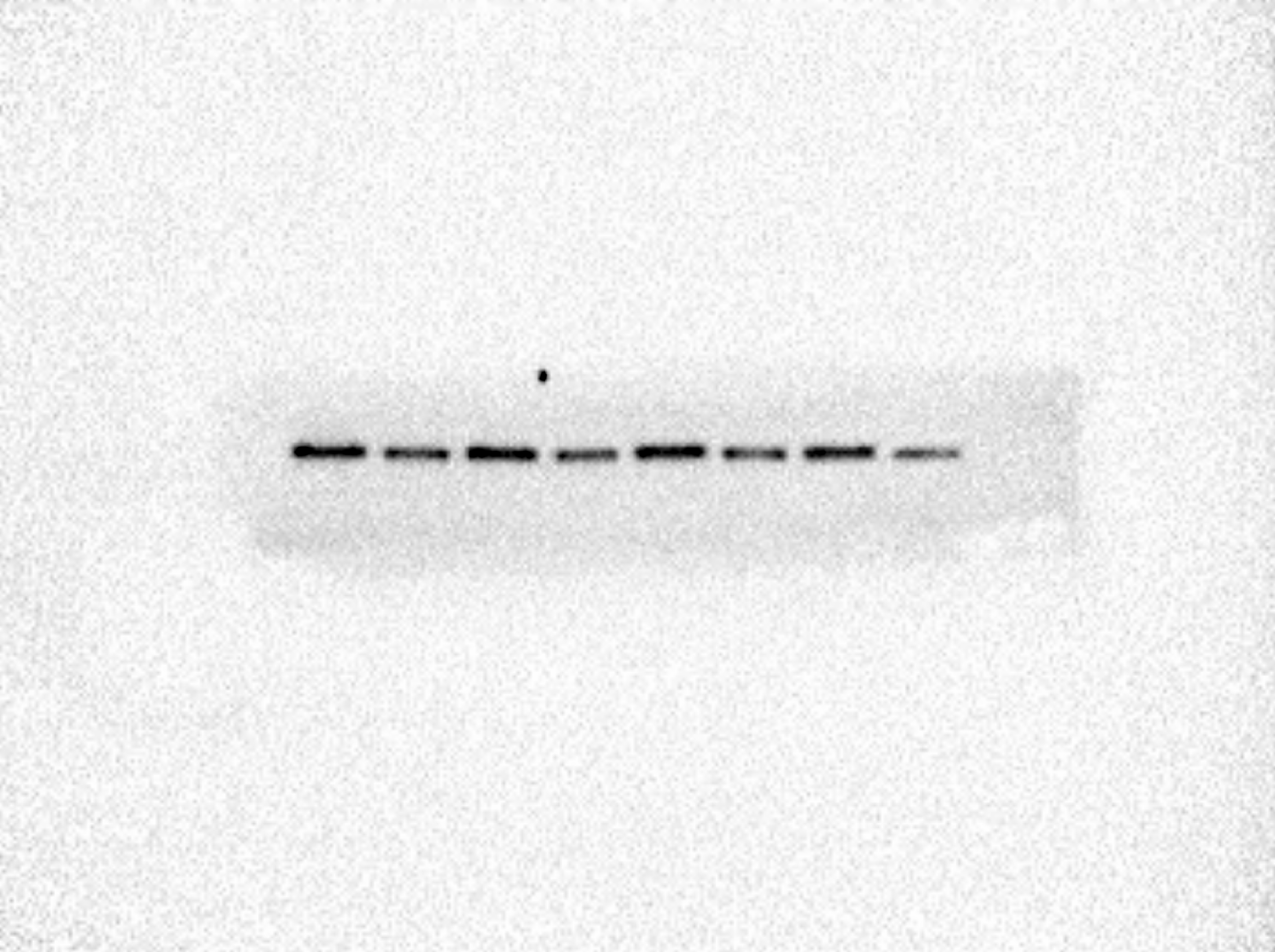

Supplement: Supplemental Information 46 — The expression of the key protein CyclinB1 in the G2 phase of the cell cycle by western blotting after cell transfection. [file peerj-09-10820-s046.jpg]
