# Supplementary material for: Abnormal expression of HOXD11 promotes the malignant behavior of glioma cells and leads to poor prognosis of glioma patients
Source: PeerJ. 2021 Feb 8;9:e10820. doi: 10.7717/peerj.10820 (PMC7877241; doi:10.7717/peerj.10820)
Supplement: Supplemental Information 36 — The cell cycle distribution ratio of sample No. 1 in the negative control group by flow cytometry after cell transfection. [file peerj-09-10820-s036.pdf]

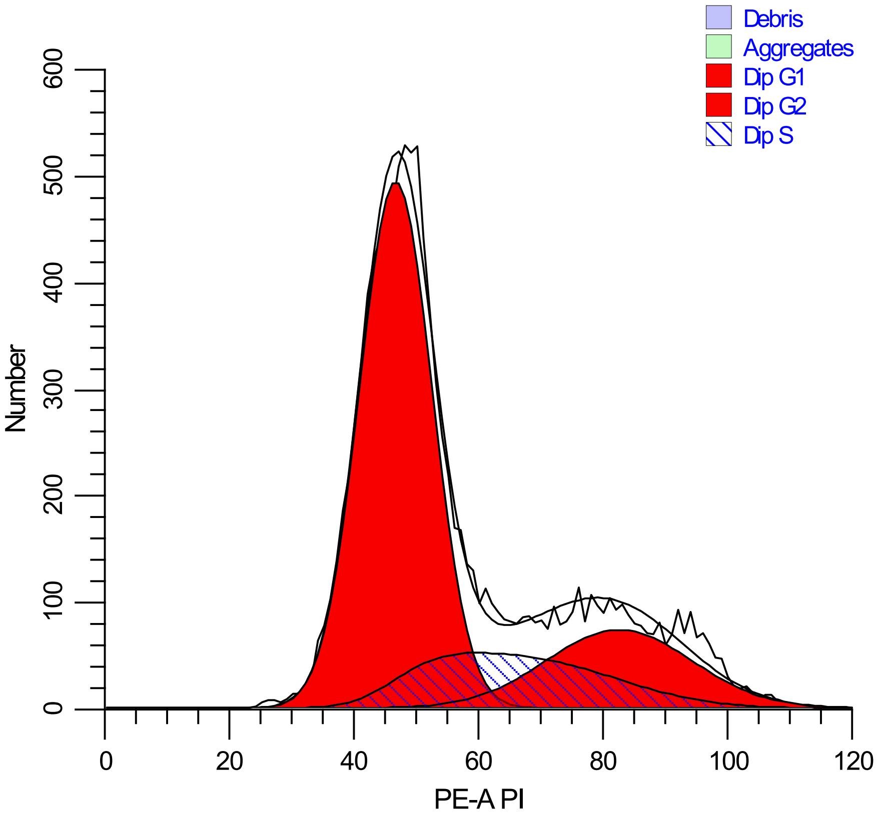

File analyzed: 001_NC-6_006.fcs
Date analyzed: 11-Aug-2020
Model: 1DA0n_DSF
Analysis type: Manual analysis
Auto Linearity: No

Ploidy Mode: First cycle is diploid

Diploid: 100.00 %
Dip G1: 63.96 % at 46.53
Dip G2: 18.99 % at 82.44
Dip S: 17.05 % G2/G1: 1.77
%CV: 12.57

Total S-Phase: 17.05 %
Total B.A.D.: 0.00 %

Debris: 0.08 %
Aggregates: 0.00 %
Modeled events: 11357
All cycle events: 11348
Cycle events per channel: 307
RCS: 1.716

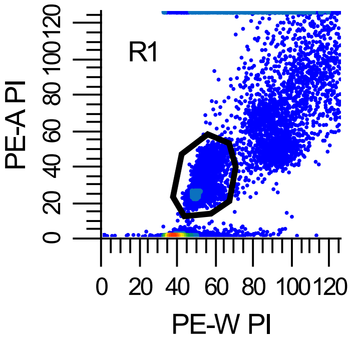

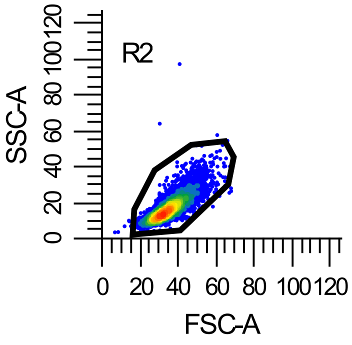

ModFit LT V4.1.7(Win)
