# Supplementary material for: Abnormal expression of HOXD11 promotes the malignant behavior of glioma cells and leads to poor prognosis of glioma patients
Source: PeerJ. 2021 Feb 8;9:e10820. doi: 10.7717/peerj.10820 (PMC7877241; doi:10.7717/peerj.10820)
Supplement: Supplemental Information 37 — The cell cycle distribution ratio of sample No. 4 in the HOXD11 gene silencing group by flow cytometry after cell transfection. [file peerj-09-10820-s037.pdf]

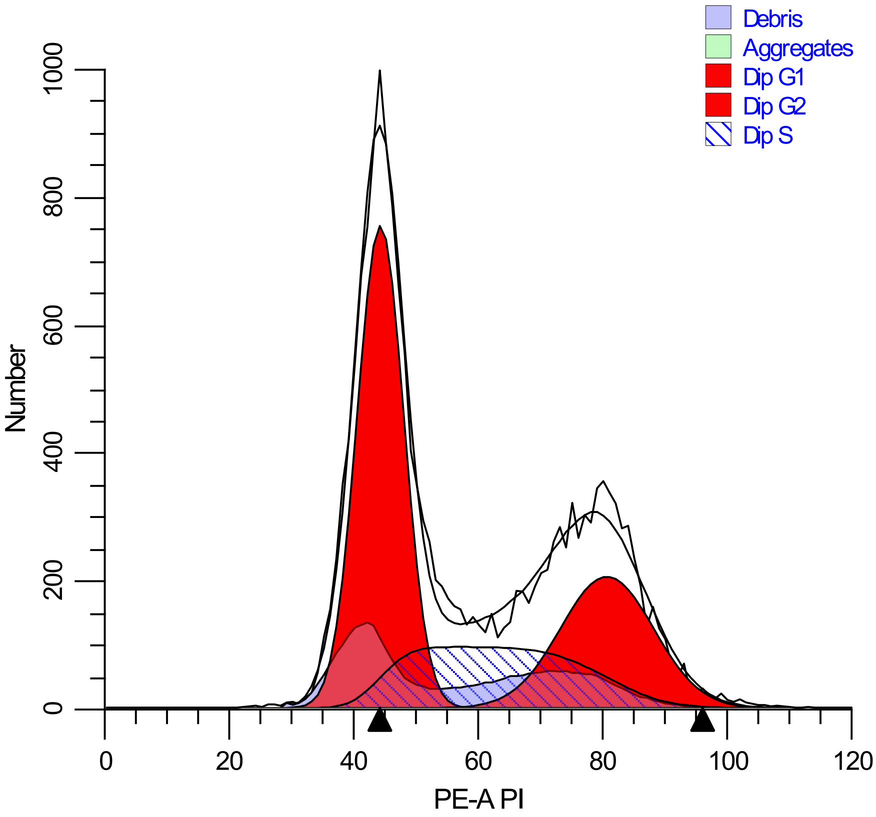

File analyzed: S2_S2-4_019.fcs
Date analyzed: 11-Aug-2020
Model: 1DA0n_DSF
Analysis type: Manual analysis
Auto Linearity: No

Ploidy Mode: First cycle is diploid

Diploid: 100.00 %
Dip G1: 49.17 % at 44.10
Dip G2: 26.71 % at 80.58
Dip S: 24.12 %   G2/G1: 1.83
%CV: 8.49

Total S-Phase: 24.12 %
Total B.A.D.: 16.62 %

Debris: 17.62 %
Aggregates: 0.08 %
Modeled events: 17581
All cycle events: 14470
Cycle events per channel: 386
RCS: 2.405

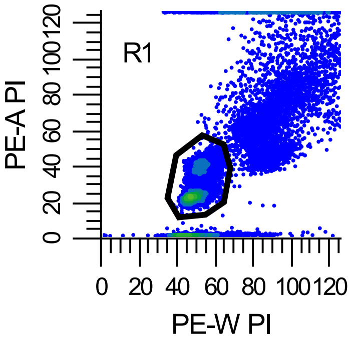

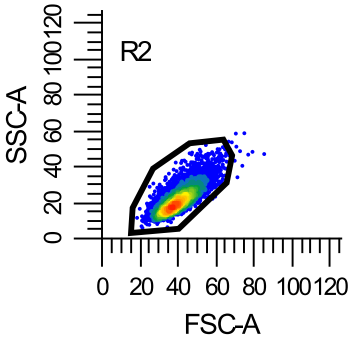

ModFit LT V4.1.7(Win)
