# Supplementary material for: Abnormal expression of HOXD11 promotes the malignant behavior of glioma cells and leads to poor prognosis of glioma patients
Source: PeerJ. 2021 Feb 8;9:e10820. doi: 10.7717/peerj.10820 (PMC7877241; doi:10.7717/peerj.10820)
Supplement: Supplemental Information 38 — The cell cycle distribution ratio of sample No. 5 in the HOXD11 gene silencing group by flow cytometry after cell transfection. [file peerj-09-10820-s038.pdf]

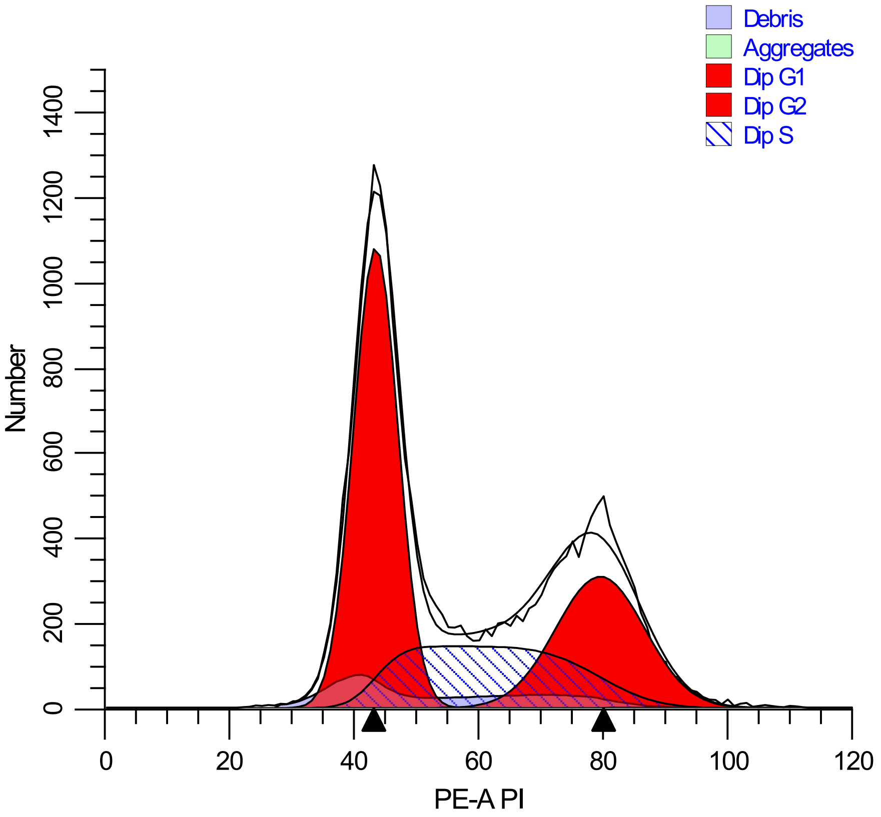

File analyzed: S2_S2-3_018.fcs
Date analyzed: 11-Aug-2020
Model: 1DA0n_DSF
Analysis type: Manual analysis
Auto Linearity: No

Ploidy Mode: First cycle is diploid

Diploid: 100.00 %
Dip G1: 47.43 % at 43.31
Dip G2: 26.99 % at 79.43
Dip S: 25.59 % G2/G1: 1.83
%CV: 8.24

Total S-Phase: 25.59 %
Total B.A.D.: 7.85 %

Debris: 8.53 %
Aggregates: 0.11 %
Modeled events: 22396
All cycle events: 20461
Cycle events per channel: 551
RCS: 1.863

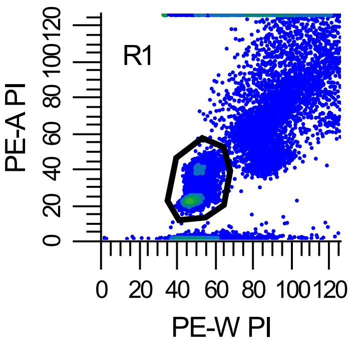

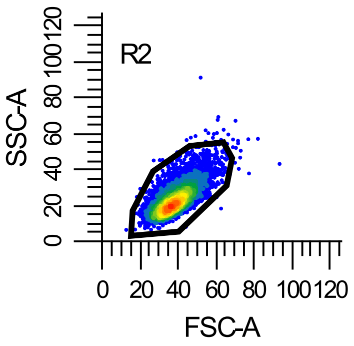

ModFit LT V4.1.7(Win)
