# Supplementary material for: Abnormal expression of HOXD11 promotes the malignant behavior of glioma cells and leads to poor prognosis of glioma patients
Source: PeerJ. 2021 Feb 8;9:e10820. doi: 10.7717/peerj.10820 (PMC7877241; doi:10.7717/peerj.10820)
Supplement: Supplemental Information 39 — The cell cycle distribution ratio of sample No. 2 in the negative control group by flow cytometry after cell transfection. [file peerj-09-10820-s039.pdf]

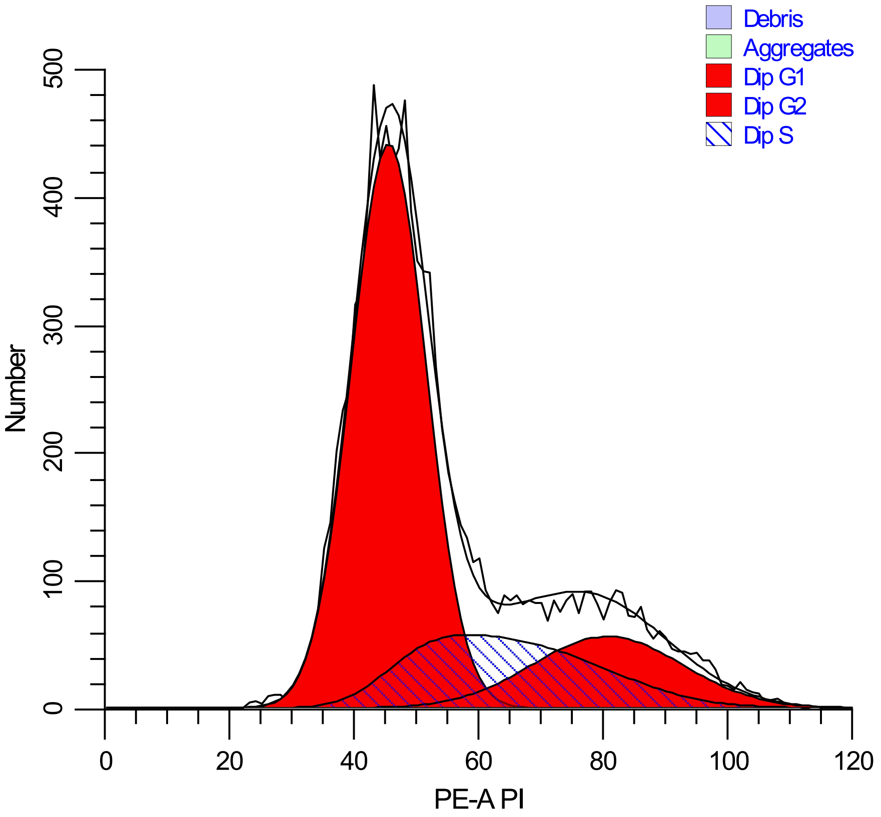

File analyzed: 001_NC-8_008.fcs
Date analyzed: 11-Aug-2020
Model: 1DA0n_DSF
Analysis type: Manual analysis
Auto Linearity: No

Ploidy Mode: First cycle is diploid

Diploid: 100.00 %
Dip G1: 63.69 % at 45.40
Dip G2: 16.11 % at 80.56
Dip S: 20.20 % G2/G1: 1.77
%CV: 13.30

Total S-Phase: 20.20 %
Total B.A.D.: 0.00 %

Debris: 0.07 %
Aggregates: 0.00 %
Modeled events: 10512
All cycle events: 10505
Cycle events per channel: 290
RCS: 1.246

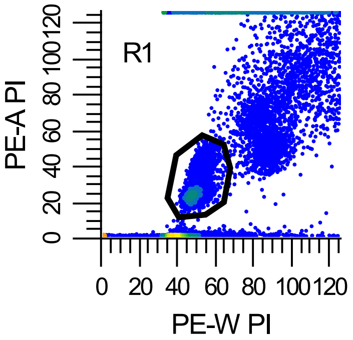

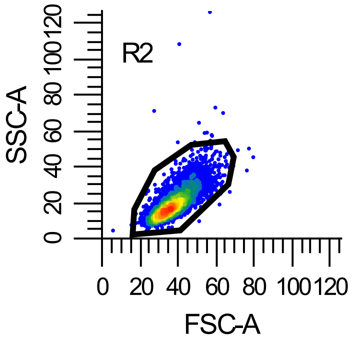

ModFit LT V4.1.7(Win)
