# Supplementary material for: Abnormal expression of HOXD11 promotes the malignant behavior of glioma cells and leads to poor prognosis of glioma patients
Source: PeerJ. 2021 Feb 8;9:e10820. doi: 10.7717/peerj.10820 (PMC7877241; doi:10.7717/peerj.10820)
Supplement: Supplemental Information 41 — The cell cycle distribution ratio of sample No. 6 in the HOXD11 gene silencing group by flow cytometry after cell transfection. [file peerj-09-10820-s041.pdf]

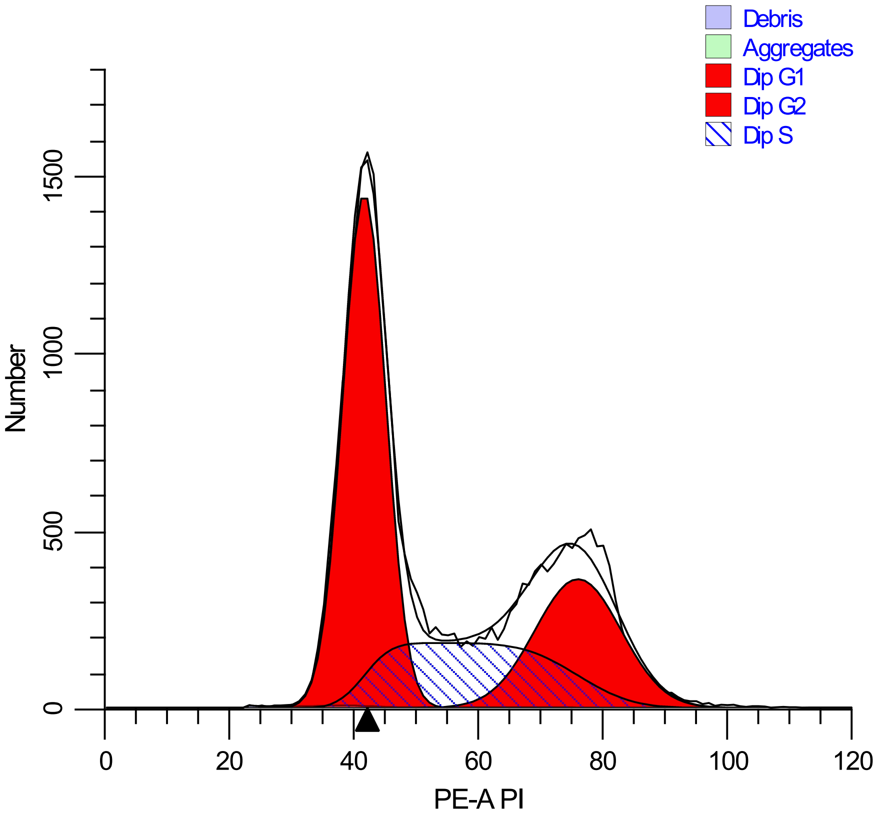

File analyzed: S2_S2-2_017.fcs
Date analyzed: 11-Aug-2020
Model: 1DA0n_DSF
Analysis type: Manual analysis
Auto Linearity: No

Ploidy Mode: First cycle is diploid

Diploid: 100.00 %
Dip G1: 49.98 % at 41.50
Dip G2: 24.96 % at 75.91
Dip S: 25.07 % G2/G1: 1.83
%CV: 8.32

Total S-Phase: 25.07 %
Total B.A.D.: 0.51 %

Debris: 0.75 %
Aggregates: 0.09 %
Modeled events: 25426
All cycle events: 25212
Cycle events per channel: 712
RCS: 2.576

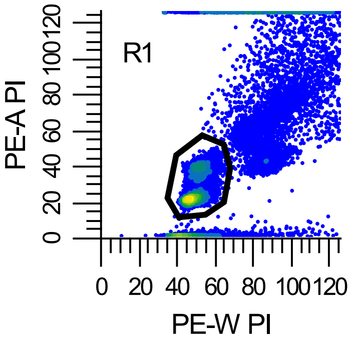

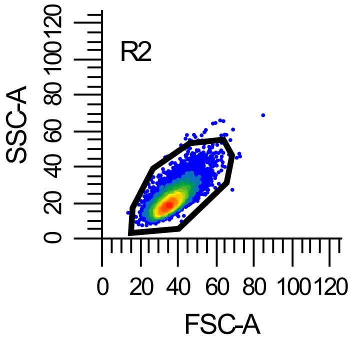

ModFit LT V4.1.7(Win)
